# Semantic Anchors Facilitate Task Encoding in Continual Learning

**DOI:** 10.1162/OPMI.a.28

**Published:** 2025-09-09

**Authors:** Mina Habibi, Pieter Verbeke, Mehdi Senoussi, Senne Braem

**Affiliations:** Department of Experimental Psychology, Ghent University, Ghent, Belgium; Department of Applied Informatics, Howest University of Applied Sciences, Kortrijk, Belgium; CLLE, Université Toulouse Jean Jaurès, CNRS, Toulouse, France

**Keywords:** continual learning, task representations, task separation, semantic labeling, reinforcement learning

## Abstract

Humans are remarkably efficient at learning new tasks, in large part by relying on the integration of previously learned knowledge. However, research on task learning typically focuses on the learning of abstract task rules on minimalist stimuli, to study behavior independent of the learning history that humans come equipped with (i.e., semantic knowledge). In contrast, several theories suggest that the use of semantic knowledge and labels may help the learning of new task information. Here, we tested whether providing existing, semantically rich task embeddings and response labels allowed for more robust task rule encoding and less (catastrophic) forgetting and interference. Our results show that providing semantically rich task settings and response labels resulted in less task forgetting (Experiment 1), both when using pictorial symbols or words as labels (Experiment 2), or when contrasted with visually matched shape labels without inherent meaning (Experiment 4). Using a subsequent value-based decision-making task and reinforcement learning modeling (Experiment 3), we demonstrate how the learned embedding of novel stimuli in semantically rich, representations, further allowed for a more efficient, feature-specific processing when learning new task information. Finally, using artificial recurrent neural networks fitted to our participants’ task performance, we found that task separation during learning was more predictive of learning and task performance in the semantically rich conditions. Together, our findings show the benefit of using semantically rich task rules and response labels during novel task learning, thereby offering important insights into why humans excel in continual learning and are less susceptible to catastrophic forgetting compared to most artificial agents.

## INTRODUCTION

Humans have the exceptional ability to continually learn different tasks, skills, and knowledge throughout their lifetime, which remains a substantial challenge in artificial intelligence (AI; Kudithipudi et al., 2022). Throughout their lifetimes, humans build on the accumulated knowledge and expertise of themselves and others. Forgetting previously learned skills, tasks, and knowledge, or experiencing strong interference between them while learning new ones, would be nothing short of catastrophic. Without continual learning, a surgeon would forget one surgical procedure after learning a new technique, an engineer would discard the fundamentals of structural design to adopt innovative architectural methods, and a student would wipe away third-grade knowledge upon moving to fourth grade.

In contrast, AI systems face significant challenges in continual learning—struggling with the ability to integrate new information without disrupting previously acquired knowledge (Chen & Liu, 2018; Flesch et al., 2022, 2023; van de Ven et al., 2024; Yang et al., 2019). This phenomenon, also known as catastrophic forgetting, is a well-documented issue in artificial neural networks but seems less prevalent in human learning. Even the most advanced AI models and algorithms that outperform humans in specific tasks, such as playing chess or Atari games, struggle with catastrophic forgetting (Mnih et al., 2015, 2016). During recent years different continual learning algorithms were developed inspired by cognitive neuroscience such as algorithms using context-dependent processing, episodic replay, or plasticity techniques (Kudithipudi et al., 2022; van de Ven et al., 2024). However, one possible reason for the remaining discrepancy between human and AI continual learning is the way humans utilize semantic knowledge and labels to anchor new information within their existing frameworks of understanding (Giallanza et al., 2024; Kaplan, & Murphy, 2000; Keil, 1979; Lupyan, 2016; Saxe et al., 2019). By exploring the potential of semantic anchors in task encoding, we aim to uncover mechanisms behind human continual learning, shedding light on the human cognitive mechanisms that enable individuals to quickly learn new tasks without forgetting old ones.

To understand the cognitive processes of task learning, most cognitive science studies focused on the various components that comprise task knowledge, including stimulus, response, contexts, outcomes, and the rules that convert input to output. These components are thought to be encoded and stored in the brain as task representations (Asaad et al., 2000; Bhandari et al., 2025; Bustos et al., 2024; Cole et al., 2016; Freedman et al., 2001; Kikumoto et al., 2022; Rangel et al., 2023; Schumacher & Hazeltine, 2016; Woolgar et al., 2011, 2015, 2016). Different studies have shown how different task components such as visual and semantic similarity of stimuli (Yoo et al., 2023), conceptual similarity (Bustos et al., 2024), task structure (flat vs. hierarchical; Bhandari et al., 2025; Verbeke, & Verguts 2025) and training regime (Blocked vs. Interleaved; Flesch et al., 2018, 2022, 2023) can influence the development of task representations and learning outcomes. A recurring idea in this literature, is that the building of more separated task representations is particularly helpful for multi-task learning and situations in which we need to perform different learned tasks in parallel or switch back and forth between them with minimal interference (Garner & Dux, 2015, 2023; Musslick & Cohen, 2021).

Beyond the various components of task knowledge such as stimulus, response, context, and task structure that influence the learning of task representations, language is also thought to play a crucial role in learning of task representations (Dove et al., 2022; Henningsen-Schomers et al., 2023; e.g., the Sapir-Whorf hypothesis; Perlovsky, 2009; Sapir, 1949; Whorf, 2012), as studies have demonstrated the influence of language on thought and cognition (Imai et al., 2016; Li & Gleitman, 2002; Luo et al., 2023), visual processing and perception (Boutonnet & Lupyan, 2015; de Vries et al., 2017; Lupyan et al., 2020), categorization (Perry & Lupyan, 2014; Zettersten et al., 2024), memory, concept perception and object representation (Lupyan, 2008, 2012a, 2012b). Using words to identify concrete objects influences how we learn new categories, remember and reason about familiar ones, and even impacts our basic visual processing (Boutonnet & Lupyan, 2015; de Vries et al., 2017; Lupyan et al., 2020). When objects are represented through language, these representations seem more distinct—more categorical—compared to representations triggered without language (Henningsen-Schomers et al., 2023). A connectionist model of “language-augmented thought” provides a computational explanation of how labeling can enhance both cognitive and perceptual processes (Lupyan, 2012a, 2016). However, despite this strong evidence of language’s broad impact, the role of semantic memory and knowledge in continual learning has received comparatively less attention (Davis, & Yee, 2021; Giallanza et al., 2024; Lupyan, 2016).

In most laboratory settings, task learning studies or task switching studies employ abstract and non-tangible stimuli that do not or barely rely on participants’ existing semantic knowledge and memory (Abrahamse et al., 2016; Collins & Frank, 2013; Kiesel et al., 2010; Vandierendonck et al., 2010; Xie & Mack, 2024). These highly controlled settings allow researchers to isolate specific cognitive processes, minimizing confounding factors. However, in the context of continual learning, we believe prior knowledge is not just a background variable but an active factor that facilitates the integration of new information and helps prevent interference between tasks. In contrast to artificial laboratory settings, real-life learning typically relies on pre-existing semantic knowledge and memories about the world. For example, when participants would be asked to categorize whether new, fictional animals can inhabit a tropical forest or a boreal forest, they already possess semantic knowledge about these ecosystems, including information about vegetation, average temperature, rainfall, and humidity. In this example, words (tropical vs. boreal forest) are considered similar to other sensory inputs, and serve as signals for understanding and contribute to the development of our mental collection of concepts (Lupyan & Lewis, 2019). This pre-existing knowledge enables participants to integrate new information about the fictional animal with their pre-existing understanding of the forest environments, and may help building separate task representations more quickly. Namely, there are reasons to believe that linking new stimuli to existing semantic structures, can help guide task formation through the faster contextualization of this task-relevant information (Giallanza et al., 2024; Hodel et al., 2022; Lupyan, 2012b) and alignment of both conceptual systems (e.g., Roads & Love, 2020; Sucholutsky et al., 2023).

### Overview of the Current Experiments

In this study, across four experiments, we set out to systematically investigate how semantically informed task rules and response labels contribute to the development of task representations by enhancing task encoding and reducing task interference during continual learning. In each of our experiments, participants needed to learn, through trial and error, to differentially categorize the same set of insects across three different tasks (see also, Mack et al., 2016). Namely, they were told that they were “space biologists” tasked with categorizing a new species of insects and determining for each insect whether it can thrive in a specific environment. Depending on the task, participants had to learn to focus on one of the three different features that varied across insects: the thickness of their legs, the thickness of their antennae, or the shape of their mandibles. Critically, we used two different cover stories to give meaning to these three separate tasks. In the semantic labeling condition, we provided a semantically meaningful embedding space by asking participants to categorize insects along three different environmental factors known to affect animal survival (e.g., Warm vs. Cold, Rural vs. Urban, and High vs. Low altitude). Conversely, in the no-labeling condition, participants were always faced with one of three differently colored planets (i.e., three tasks), and had to determine, for each planet, whether the insect could live there, or not (but used a different manipulation in Experiment 4). Importantly, in both versions, the tasks were exactly the same, and the participants had to learn per environmental factor or planet to focus on one of the three bug features to generate a correct response.

In Experiment 1, we systematically manipulated these task labels (Semantic-labeling vs. No-labeling), but also manipulated presentation order (Context-first vs. Stimulus-first). In most task learning or task-switching studies, the task cue or context cue is usually presented first, allowing participants to develop an action plan based on a clear, predetermined goal (Davachi, 2006; Eichenbaum et al., 2007; Jiang et al., 2020; Place et al., 2016; Smith & Bulkin, 2014). However, in everyday life, tasks often unfold in more varied ways—such as deciding what to cook after looking through available ingredients. Similarly, task-switching studies have demonstrated that task-relevant stimuli can sometimes function as cues triggering different tasks (Koch & Allport, 2006; Schmidt & Liefooghe, 2016; Waszak, 2010). Therefore, introducing the stimulus first may encourage a more flexible approach to task learning, promoting a readiness to adapt to various task contexts, enhancing continual learning. To test the effects of both semantic labeling and presentation order, participants were assigned to one of four groups: Context-first No-label, Context-first Semantic-label, Stimulus-first No-label, and Stimulus-first Semantic-label. Participants’ performance was evaluated during both the learning phase, as well as a subsequent test phase where they needed to switch back and forth between these different tasks, and no more feedback was given. We predicted that semantically rich response labels and learning environments where stimuli are presented first would help enhance the separability of task representations and robustness of task encoding, resulting in a reduced susceptibility to forgetting and interference.

Experiment 2 served as a replication and control experiment of Experiment 1, to study whether we could replicate the findings for the condition where we predicted the least interference (Stimulus-first Semantic-label), and control whether similar effects were observed when using written words instead of symbolic icons (i.e., written words for Warm and Cold labels instead of symbols; similar to the No-label condition in Experiment 1 that used Yes/No words).

In Experiment 3, we investigated whether the richer encoding and more separated task representations from the Semantic-label condition would also help participants learn new relations in a subsequent value-based decision-making task (Farashahi & Soltani, 2021), by studying behavior and attentional weighting parameters using a dedicated reinforcement learning models.

Finally, in Experiment 4, we tried to determine whether the observed benefits in the Semantic-labeling condition, as shown in Experiment 1, were driven by the semantically rich task embedding or merely by providing separate response labels per task. Therefore, we developed another control condition and compared semantically informative labels to shape labels (e.g., triangle vs. star), which lacked inherent semantic meaning.

To preview our main findings, we observed that, compared to the ‘No-label’ condition, providing distinct labels in the ‘Semantic-label’ condition for each task resulted in better retained task representations, thereby enhancing the robustness of continual learning and minimizing task interference during the test phase, where participants were required to switch between the three learned tasks. This effect was especially pronounced when participants encountered the stimulus before the contextual task information (Experiment 1). This effect held regardless of whether words or symbols were used as semantic labels (Experiment 2), and was also observed when comparing semantic labeling to a version that provided separate, concrete response labels that were semantically meaningless (Experiment 4). In Experiment 3, we demonstrated that access to semantic labels during task learning helped participants in a subsequent, independent value-based decision-making task, where successful decision-making required reconfiguring learned relevant stimulus features.

In the final part of our paper, we present artificial neural network simulations of participants’ behavior as an explorative test of the idea that semantic labeling helped with the construction of more separate task representations. An increasingly popular approach to analyse human behavior are artificial neural networks (Eckstein et al., 2023; Ma & Peters, 2020). Despite their more limited interpretability due to the large number of parameters, they often a good fit of human performance (Ji-An et al., 2024), and investigating the activation patterns in the hidden layer(s) can allow us to explore how a neural network organizes task representations for performance (Verbeke & Verguts, 2025). Here, we similarly used artificial neural networks to obtain new summary statistics of our participants’ behavior that go beyond average task accuracy. Specifically, by studying the similarities across every stimulus-task combination in the hidden layer activation of a model trained on participants’ responses, we derived a potential measure of task separation (dissimilarity between tasks) and task specialization (similarity to a normative task similarity matrix within tasks), and studied the effect of labeling and their relation to task learning. We provide tentative support for the usefulness of these measures and the interpretation that semantic labeling reinforced task separation, which we believe can inspire future research in this domain.

## EXPERIMENT 1

### Methods

#### Participants.

We recruited 297 participants via Prolific from the US population, with the additional restriction of having an approval rate higher than 95% (i.e., the person has not been rejected after performing in other research studies for more than 95% of the studies they participated in). Before starting the study, participants provided consent in accordance with the local ethics guidelines of Ghent University. We removed data from participants based on the following criteria: a mean response time lower than 300 milliseconds, not responding to more than one-third of the trials, or pressing the same response key continuously for more than 90% of the trials, during one of the blocks. Additionally, we only considered participants with an accuracy greater than 57% in the second half of the learning phase, which is the threshold to score significantly above chance according to a chi-squared test. Data collection continued until we had at least 55 participants per group, which is sufficient for detecting a group difference with a statistical power of 80% and medium effect size of Cohen’s d = .5 (required sample size is 51 per group according to G*Power), and similar or more than other between-subject studies on task learning from our lab (Held, Vermeylen, Dignath, et al., 2024; Held, Vermeylen, Krebs, et al., 2024) or other labs (Flesch et al., 2018; Longman et al., 2024). After applying these criteria, 32 participants were removed (10.8 %), resulting in a final sample of 265 participants, aged between 18 and 35 years. Participants were randomly assigned to one of four experimental groups: *Context-first No-label*: Mean age of 28.32 ± 4.39 years (30 female, 31 male, and 1 others); *Context-first Semantic-label*: Mean age of 29.28 ± 4.59 years (32 female, 36 male); *Stimulus-first No-label*: Mean age of 28.47 ± 6.67 years (30 female, 36 male, and 4 others); *Stimulus-first Semantic-label*: Mean age of 28.06 ± 4.74 years (29 female, 32 male, and 4 others). There were no significant differences in age or gender across the different groups. Participants were also invited to complete the test phase again on a second day to examine the effects of sleep (consolidation) on task performance. However, the data of this second phase are not discussed here to keep the focus of the paper on the study of semantic labeling and stimulus-context presentation order.

#### Stimuli and Materials.

**Figure 1.**
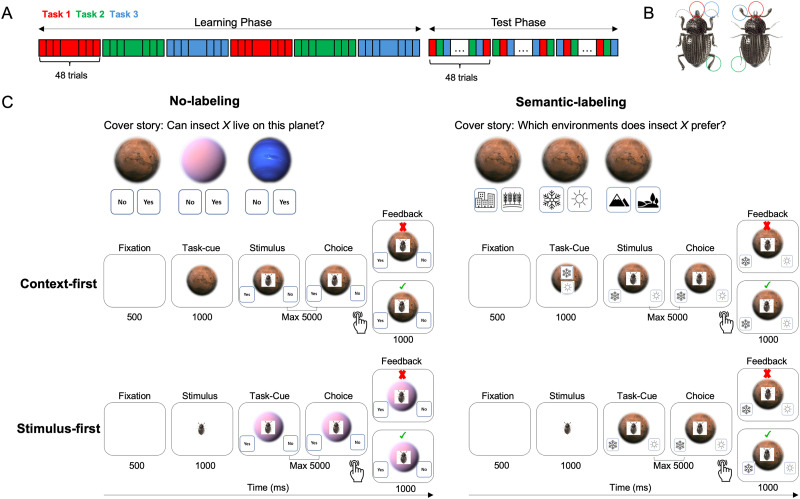
**Experiment 1 Task Design.** **(A)** Experiments 1 used a between-group design, with four groups undergoing 288 learning trials (48 trials per block; 96 trials per task, spread over two blocks per task) followed by 144 interleaved test trials (three blocks, with 48 trials per task). The order of tasks was counterbalanced among participants. **(B)** The insect stimuli systematically varied along three features: leg thickness (thick or thin), antennae thickness (thick or thin), and mandible type (shovel or pincer). **(C)** Participants took on the role of biologists working across three distinct environments. In the No-labeling groups (*Context-first No-label* and *Stimulus-first No-label*), participants’ spaceship landed on one of three planets (Pink, Blue, or Brown), and they had to decide whether the insect shown on the screen could survive on that planet. The planet’s color served as a cue, indicating which feature—legs, antennae, or mandibles—was relevant for the task. In the Semantic-labeling groups (*Context-first Semantic-label* and *Stimulus-first Semantic-label*), participants stayed on the same planet but had to determine which environment on the planet the insect preferred for living. The environment label indicated the relevant feature. In both No-label and Semantic-label conditions, participants figured out the insect’s preferred environment through trial and error. Each trial started with a fixation phase. In the Context-first groups, this was followed by the presentation of a task cue, the stimulus, response labels, and a feedback phase, in that order. In the Stimulus-first groups, the fixation phase was followed by the stimulus presentation, then the task response labels as task cues, and finally the feedback. The test trials followed the same structure but without feedback. The location of the response keys (for example, “Yes” or “No”) was shuffled on a trial-by-trial basis.

Our stimuli consisted of eight images (see also Mack et al., 2016). The insects were composed of a factorial combination of three features: leg, mandible (i.e., jaw), and antennae, each of which had two values (thick vs. thin legs, thick vs. thin antennae, and shovel vs. pincer mandible), resulting in eight unique stimuli (see Figure 1B). The same stimuli were used across the three tasks in both the learning and testing phases. Each insect was shown twelve times per task during the learning phase and six times during the test phase.

#### Task and Procedure.

The general structure was the same for all participants. Specifically, participants were instructed to categorize each stimulus (insect) based on the task-cue (context). The instruction did not explicitly cue participants for the relevant dimension, so participants had to learn the three tasks through trial and error (e.g., Task 1 required consideration of leg thickness, Task 2 required consideration of antennae thickness, and Task 3 required attention to the shape of the mandible). All participants performed six blocks of 48 trials each during the learning phase to learn the three different tasks. The order in which the three tasks were presented was randomized for the first three blocks, and repeated for the next three (i.e., Task 1, Task 2, Task 3, Task 1, Task 2, Task 3; see Figure 1A). This semi-blocked design better reflects naturalistic learning settings and aligns with classic approaches in the literature on blocked vs. interleaved (or massed vs. spaced) training (e.g., Carvalho & Goldstone, 2014, 2015; Mack et al., 2020; Schmidt & Bjork, 1992; Shea & Morgan, 1979). Immediately following the learning phase, participants proceeded to the test phase, which consisted of three blocks of 48 trials each (Figure 1A). The test phase differed from the learning phase in that it provided no feedback and intermixed the three tasks within each block. Participants were allowed a self-paced break of up to two minutes after each block.

Importantly, the *Semantic-label* versus *No-label* groups received different cover stories. Specifically, the Semantic-label condition (comprising both *Context-first Semantic-label* and *Stimulus-first Semantic-label* groups) immersed participants in an imaginative scenario, casting them as biologists on a space journey with a collection of insects. Upon landing on a new planet, they were tasked with determining which environmental condition the insect on screen preferred. They were presented with one of three environmental conditions that are also relevant for categorizing optimal living conditions for animals more broadly, such as “warm vs. cold,” “high vs. low,” and “urban vs. rural” as response options (Mack et al., 2020). In this case, the response labels correspond to pre-existing, meaningful conceptual dimensions that are naturally far apart in conceptual space (e.g., “warm” and “cold” are conceptually distinct, not arbitrary). Thus, the manipulation introduces pre-existing semantic structures into the task to scaffold learning. In the *No-label* condition (comprising both *Context-first No-label* and *Stimulus-first No-label* groups), however, participants again assumed the role of space biologists, but now travelled between three different planets (blue, pink, or brown), and their task was to determine whether the insect on the screen could survive on that particular planet or not (Figure 1C). In contrast to the environmental conditions in the Semantic-label group, these three planets were unknown in general, but also unfamiliar for categorizing animals in particular.

Orthogonal to the Semantic-label versus No-label manipulation, participants could be further categorized in a Context-first versus Stimulus-first group. In the *Context-first* groups (comprising both *Context-first Semantic-label* and *Context-first No-label* groups), each trial began with a fixation cross displayed at the screen’s center for 500 milliseconds, followed by a planet image that appeared for 1000 milliseconds, establishing the task context. In the *Semantic-label* group, this planet image was always of the same color, but further contained the categorization cues relevant for the task they would have to perform (Figure 1C). In the *No-label* group, this planet presentation could be one of the three differently colored planets, depending on which task they would have to perform (Figure 1C). Next, an insect appeared at the center of the planet, with response labels presented on the lower left and right sides of the planet, with their locations randomized and counterbalanced across trials. In the *Stimulus-first* groups, the fixation cross was first followed by presentation of the stimulus (insect) for 1000 milliseconds. Following this stimulus presentation, the task information (i.e., contextual cue) was added. That is, the insect was now presented on the background of the relevant planet (i.e., always the same color in the Semantic-label groups; one out of three colors in the No-label groups), and flanked by the relevant response labels on the lower left and right sides of the planet (i.e., one of three task-relevant labels in the Semantic-label groups; Yes versus No response in the No-label groups), with their locations randomized and counterbalanced across trials.

In all groups, participants conveyed their responses by pressing the D or K buttons on the keyboard corresponding to the left or right response labels, respectively, within a 5000-millisecond timeframe. Feedback was provided based on their accuracy, with a green tick for correct trials and a red cross for incorrect or non-response trials displayed above the planet image for 1000 milliseconds. Timings in the test phases mirrored those of the learning blocks, with the exception that no feedback was provided.

#### Data Analysis.

We first computed the average accuracies for each block of the learning and test phases to visualize the learning effect (learning phase), and the retained task knowledge after removing task feedback (test phase). Next, we averaged accuracies across every three blocks, as all three tasks were repeated every three blocks. We were particularly interested in the potential drop in accuracy (i.e., forgetting) from learning to test, so we focused on the change in mean accuracy of Blocks 4, 5, and 6 during the learning phase compared to the combined mean accuracy of Blocks 7, 8, and 9 during the test phase. Statistical analyses were conducted using repeated measures ANOVA with the factors Time (Learning vs. Test) as a within-subjects factor, and Labeling (Semantic-labeling vs. No-labeling) and Order (Stimulus-first vs. Context-first) as between-subjects factors.

### Results

#### Impact of Semantic Labeling vs. No Labeling and Stimulus-Context Presentation Order (Stimulus-First vs. Context-First) on Performance Accuracy.

##### Semantic Labeling Preserves Accuracy From Learning to Test, With a Stronger Impact in Stimulus-First Groups.

**Figure 2.**
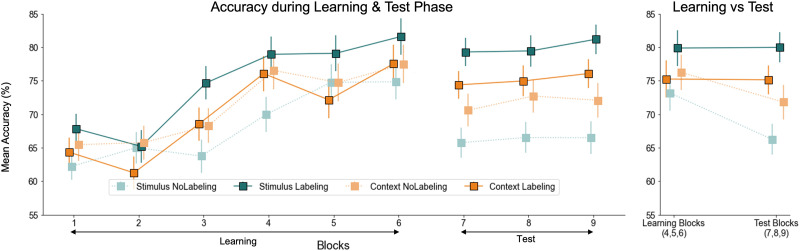
**Experiment 1 Results.** *Note*: All error bars represent the standard error of the mean (*SEM*). Learning curves and test-phase accuracy: mean accuracy is shown, averaged over 48 trials per block. On the right side of the line plot, the performance for each group is averaged over blocks 4, 5, and 6 of the learning phase and blocks 7, 8, and 9 of the test phase. During the learning phase, there were no significant differences in accuracy between groups. However, in the testing phase, the Semantic-labeling groups showed significantly higher accuracy than the No-labeling groups, reflecting better retention of task knowledge (*p* < .001). This difference was driven by a significant decline in accuracy for the No-labeling groups from learning to testing (*p* < .001). Additionally, the difference between conditions was significant only in the Stimulus-first group (*p* < .001), but not in the Context-first group. The stars indicate levels of statistical significance: *p* < .05 (*), *p* < .01 (**), and *p* < .001 (***).

Our overall repeated measures ANOVA on accuracy indicated both a main effect of Time, *F*(1, 261) = 19.978, *p* < .001, *η*^2^ = 0.007 (i.e., different between the mean accuracy of blocks 4, 5, and 6, vs. mean accuracy of blocks 7, 8, and 9), and a main effect of Labeling, *F*(1, 261) = 7.494, *p* = .007, *η*^2^ = 0.026. Importantly, however, the two-way interaction between Labeling and Time was also significant, *F*(1, 261) = 19.86, *p* < .001, *η*^2^ = 0.007, indicating that task accuracy was differentially affected from learning to test, depending on whether participants were in the Semantic-labeling condition or not. Follow-up analyses indicated that the different groups did not differ in accuracy during the learning phase, depending on whether the tasks implemented Semantic-labeling or not, *F*(1, 263) = 1.88, *p* = .172, *η*^2^ = 0.007. However, the Semantic-labeling groups (Stimulus-first Semantic-label and Context-first Semantic-label) were significantly more accurate during the testing phase, *F*(1, 263) = 14.7, *p* < .001, *η*^2^ = 0.053, suggesting they were more successful at retaining task knowledge from learning to test (see Figure 2). Splitting the interaction per group type showed that accuracy significantly dropped from training to test in the No-Labeling groups (Stimulus-first No-label and Context-first No-label), *F*(1, 130) = 26.0, *p* < .001, *η*^2^ = 0.024, but not in the Semantic-labeling groups (Stimulus-first Semantic-label and Context-first Semantic-label), *F*(1, 131) = 0.00, *p* = .99, *η*^2^ = 0.000. This again suggest that only the Semantic-labeling groups retained their training accuracy over time.

Finally, no other effects reached significance, all *F*(1, 261) < 1.12, *p* > .29, except for a two-way interaction between Labeling and Order, *F*(1, 261) = 4.722, *p* = .031, *η*^2^ = 0.016, suggesting there was an overall difference in accuracy between the Semantic-labeling and No-labeling condition in the Stimulus-first groups, *F*(1, 268) = 21.9, *p* < .001, *η*^2^ = 0.001, but not the Context-first groups, *F*(1, 258) = 0.29, *p* = .591, *η*^2^ = 0.001 (Figure 2).

#### Task-Specific Forgetting Patterns and the Role of Semantic Labeling.

##### In the No-Labeling Condition, Forgetting Disproportionately Affected Earlier-Learned Tasks, Consistent With Catastrophic Interference.

To assess whether the forgetting that was observed in the No-labeling group was more pronounced in the earlier-learned tasks compared to the last learned task (i.e., Task 3), we compared accuracy between the learning phase (Blocks 4–6) and the test phase (Blocks 7–9) for Tasks 1 versus 3 or 2 versus Task 3. Specifically, we conducted repeated-measures ANOVAs with Time (Learning vs. Test) and Task (Tasks 1 vs. Task 3 or Task 2 vs. Task 3) as within-subjects factors, and Order (Context-first vs. Stimulus-first) as a between-subjects factor in the no-labeling group. Based on a catastrophic forgetting hypothesis, we expected greater declines for Tasks 1 and 2 than for Task 3.

**Figure 3.**
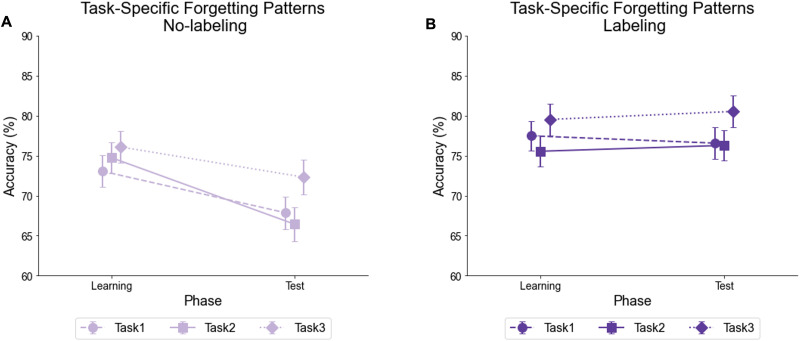
**Task-Specific Forgetting Patterns With and Without Semantic Labeling.** *Note*: All error bars represent the standard error of the mean (*SEM*). **(A)** In the No-labeling condition, Task 1 showed generally lower accuracy than Task 3 (main effect of Task, *p* = .046), and Task 2 showed a larger decline from learning to test (*Mean*-diff = 0.083, *p* < .001) than Task 3 (*Mean*-diff = 0.038, *p* = .020), with marginally lower test performance (*p* = .051), consistent with retroactive interference. While in the **(B)** Labeling condition, no evidence of task-specific forgetting was observed; performance remained stable across tasks.

For the Task 1 vs. 3 comparison, there were significant main effects of Time (*F*(1, 130) = 13.2, *p* < .001) and Task (*F*(1, 130) = 4.06, *p* = .046), indicating an overall higher accuracy for Task 3, but no significant interaction between Time and Task. For Task 2 vs. 3, however, a significant Time effect emerged (*F*(1, 130) = 21.7, *p* < .001), along with a non-significant Task × Time interaction (*p* = .058) that hinted at a differential effect of Time on Task 3 versus 2. Consistent with the idea of catastrophic forgetting, accuracy declined more for Task 2 (M_diff = 0.083, *p* < .001) than for Task 3 (M_diff = 0.038, *p* = .020; Figure 3A). A similar analysis in the Labeling group showed no effects of Time, or interactions between Time and Task, consistent with the main analyses.

These findings suggest that retroactive interference occurs for earlier-learned tasks in the No-labeling condition (Figure 3B). Although this effect seemed specific to Task 2 and not Task 1, this could be because Task 1 may still have benefited from a primacy effect.

### Discussion

Our first experiment indicated that using semantically meaningful task embeddings during continual learning significantly enhanced encoding, helped against task interference, and helped retain accuracy across the different tasks during the test phase, where efficient task-switching was required, and reduced interference between tasks was desirable. The Semantic Labeling effect seemed particularly pronounced in the Stimulus-first condition, where participants had more time to process the stimulus and associate each insect feature with different labels. However, this was not consolidated in a three-way interaction, nor did presenting the Stimulus first show an overall benefit over time in the same way Semantic labeling did.

Interestingly, while we observed a clear benefit of semantic labeling during the test phase, no performance difference emerged during the learning phase, when participants practiced the three tasks in a blocked format. This possibly reflects the reduced need for task separation under isolated learning conditions. In contrast, the test phase introduced frequent task-switching, increasing the demand for interference control. Under these conditions, richer and more distinct task representations—supported by semantic labels—became especially beneficial, helping participants manage interference and better distinguish between tasks.

Overall, these findings highlight the importance of considering both labeling strategies and temporal dynamics when designing cognitive tasks and interpreting their outcomes. Given that the effect of semantic labeling seemed most pronounced in the Stimulus-first condition, we chose to continue with this presentation order in the next experiments. In the next step, we first wanted to see whether we could replicate the broader observation that the Stimulus-first Semantic-label group showed no drop in accuracy from learning to test, while also extending our findings to written word labels instead of symbolic labels.

## EXPERIMENT 2

The objective of Experiment 2 was to replicate the results from Experiment 1 within the Stimulus-first Semantic-Label condition and to investigate whether the semantic labeling effect observed in Experiment 1 was due to the use of symbolic labels in the response-label group (e.g., sun vs. snow symbols to cue warm vs. cold environments in labeling groups) or could be observed with written word labels as well. If the observed benefit (i.e., no forgetting from training to test) was due to the underlying semantic representation rather than the visual symbols used to represent them, we should observe similar effects for both. To examine this, Experiment 2 compared the effects of *Symbolic* versus written *Word* labels within the Stimulus-first condition (Figure 3A).

### Methods

#### Participants.

A cohort of first-year psychology students (*n* = 59) from Ghent University, Belgium, was recruited via the SONA system platform. Participants provided informed consent in accordance with the local ethics guidelines of Ghent University and were aware of their right to withdraw from the study without penalty. Participants were removed based on the same performance criteria as Experiment 1 (i.e., a mean response time faster than 300 ms, not responding more than one-third of the trials, or pressing the same key continuously for more than 90% of trials, for any block; or an accuracy lower than 57% in the second half of the learning phase). The Word group comprised 29 participants (23 female, 6 male, 3 participants removed due to the low accuracy), while the Symbol group included 24 participants (18 female, 6 male, 3 participants removed due to the low accuracy). Participants were compensated with a study credit for their participation in this research. Due to a small programming error, we were unable to record the participants’ ages.

#### Stimuli and Materials.

**Figure 4.**
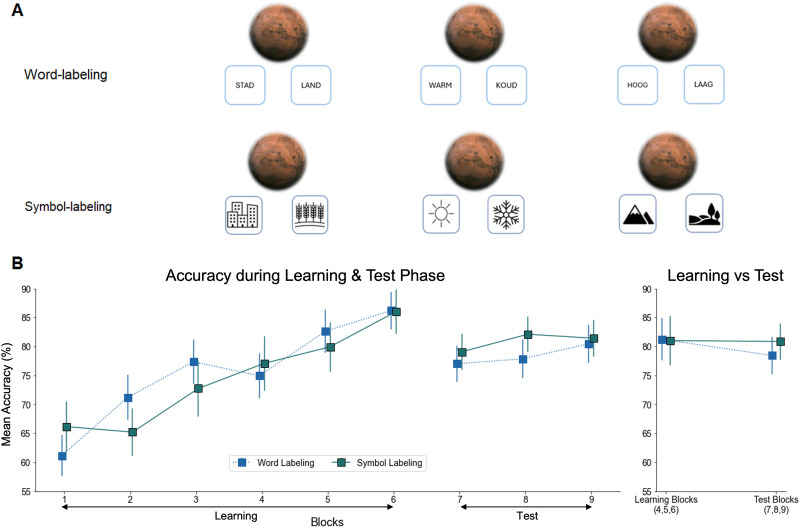
**Experiment 2 Task Design and Results.** *Note*: Error bars represent the standard error of the mean (*SEM*). **(A)** Semantic Labeling: In the Stimulus-first Symbol-label group, semantic labels were communicated using symbols, similar to Experiment 1. In the Stimulus-first Word-label group, semantic labels were conveyed through words. **(B)** Learning Curves: Mean accuracy is shown, averaged over 48 trials per block, along with test-phase accuracy. The performance across both groups is averaged over blocks 4, 5, and 6 of the training phase, and blocks 7, 8, and 9 of the test phase. Results showed no significant difference between two groups in either phase.

Stimuli and material were identical to Experiment 1, except for the labels used in the Word label group. Namely, we used the labels “STAD” and “LAND” which refer to the urban versus rural conditions in Dutch, “WARM” and “KOUD” for warm and cold, and “HOOG” and “LAAG” for high and low altitude, instead of the symbolic cues used in Experiment 1 and for the Symbol group in this experiment (Figure 4A).

#### Task and Procedure.

The procedure for both *Word-Label* and *Symbol-Label* conditions was the same as the Stimulus-first Semantic label condition in Experiment 1.

### Results

#### Impact of Symbol vs. Word Labeling on Performance.

##### Participants Retained Task Knowledge Equally Well From Learning to Test, Regardless of Whether Tasks Were Labeled With Symbols or Words.

Similar to Experiment 1, we fist computed the mean accuracy over 48 trials for each block during both the learning and test phases, after which we compared the combined mean accuracy of Blocks 4, 5, and 6 during the training phase, with that of Blocks 7, 8, and 9 for the test phase.

A mixed 2 × 2 ANOVA was conducted with Time (Train vs. Test) as a within-subjects factor, and Label (Word vs. Symbol) as a between-subjects factors. There was no significant main effect of Group, *F*(1, 51) = 0.083, *p* = .775, suggesting the type of Label (Symbol vs. Word) did not impact learning. Importantly, there was also no main effect of Time on accuracy, *F*(1, 51) = 1.485, *p* = .229, suggesting accuracy did not decrease from training to test and participants successfully retained task knowledge. Finally, the interaction between Group and Time was also not significant, *F*(1, 51) = 1.208, *p* = .277 (Figure 4B).

### Discussion

The results from Experiment 2 confirm the findings of Experiment 1 that labeling assists participants during continual learning. Specifically, Experiment 2 showed that semantic representations, whether presented as words or symbols, help participants dissociate task representations and maintain performance accuracy in the test phase. This indicates that the positive effect of semantic labeling on continual learning in Experiment 1 is independent of its symbolic format and remains consistent regardless of whether the label is a word or a symbol. Taken together, we believe our two experiments indicate that Semantic Labeling can help create more structured task representations and allow for a more efficient, separated processing of the individual stimulus features. Next, we wanted to investigate whether this effect would also benefit the learning of new tasks. Specifically, our aim was to study whether learning about insects in a more semantically structured or informed task environment would also benefit the further learning about these same insect features in a subsequent new environment, where participants needed to learn the value of separate insect features or combinations of features.

## EXPERIMENT 3

In Experiment 3, we wanted to investigate whether newly learned semantic structures could influence learning in a different task or cognitive domain. To do so, we used a reinforcement learning paradigm that allows us to not only assess whether prior semantic learning influences new task learning, but also to examine *how* it does so, specifically, whether it differentially affects participants’ attention in a novel task context.

Reinforcement learning often requires selective attention to different features for humans to flexibly allocate credit and make decisions based on relevant features in their environment (Leong et al., 2017; Radulescu et al., 2019, 2021). This phenomenon has been extensively studied and demonstrated through tasks such as the Wisconsin Card Sorting Test (Brooks et al., 2003; Nasiri et al., 2023; Somsen, 2007). Recent findings suggest that language, particularly the nameability of environmental features, plays a significant role in reinforcement learning. For instance, Radulescu et al. (2022) showed that language provides a representational basis that influences both reinforcement learning and selective attention. Building on these insights, we hypothesized that people who developed enriched and distinct task representations and semantic structures for newly learned insects (as in our Semantic-labeling group), would benefit from these in the follow up reinforcement learning task when they need to direct their attention to different features of insects (antennae, mandible, leg). Therefore, we ran a third experiment to test this hypothesis.

In reinforcement learning, when people are confronted with multi-dimensional stimuli and environments, they often adopt a feature-based learning strategy to manage complexity and dimensionality. This approach involves the learning of reward values of individual features shared across different options, which facilitates rapid learning and generalization when consistent rules are present (Dezfouli & Balleine, 2019; Farashahi et al., 2017, 2018, 2020; Farashahi & Soltani, 2021; Franklin & Frank, 2019; Schaaf et al., 2022). For instance, if a child enjoys eating green grapes, they might assume they’ll like other green fruits as well. However, this assumption is not always accurate—some green fruits, like green apples or green bananas, can be sour or bitter and unripe. Therefore, when a feature-based strategy proves unreliable (e.g., choosing fruits based on color), individuals will tend to shift to a more precise strategy, object-based learning strategy, which focuses on learning the reward values of entire stimuli rather than individual features. Object-based learning strategy allows for more accurate and reliable learning outcomes, albeit at the cost of lower generalizability (Farashahi et al., 2017, 2018, 2020; Farashahi & Soltani, 2021).

**Figure 5.**
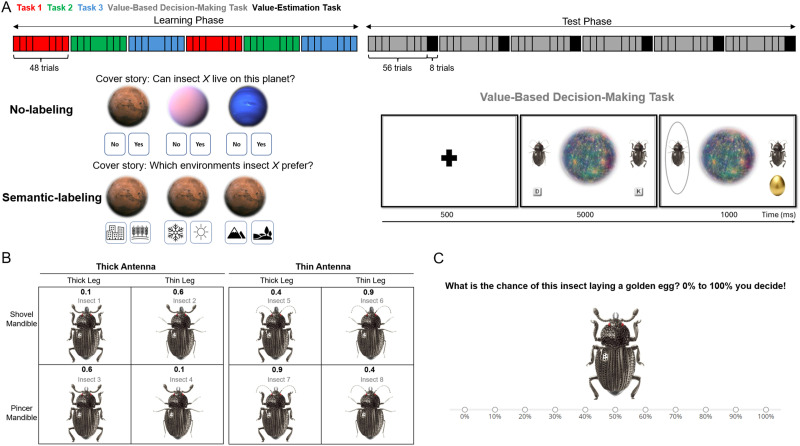
**Experiment 3 Task Design and Paradigm.** *Note*: **(A)** Experiment Timeline: In the learning phase, participants were divided into two groups: the No-labeling group (upper row) and the Semantic-labeling group (lower row). Both groups learned three tasks continuously over 6 blocks (48 trials per block, with 96 trials per task, spread across two blocks per task). The order of tasks was counterbalanced among participants. In the subsequent test phase, participants had to perform a value-based decision-making task consisting of 6 blocks of 56 trials each, where they had to decide which of the two insects presented on screen was more likely to produce a Golden Egg. At the end of each block, they also completed a value estimation task as explained in part C. **(B)** Reward Probability Table: The stimuli consisted of eight insects with three distinguishing features: legs, antennae, and mandibles. Insects with thick antennae had a lower average reward probability of 35%, compared to 65% for those with thin antennae. The thickness of the legs and the shape of the mandibles alone did not reliably indicate the insect’s value, but their combination did. For instance, the average reward probability for insects with shovel-shaped mandibles was 50%. **(C)** Value-Estimation Task: At the end of each block in the value-based decision-making task, participants performed a value estimation task. During this task, they were shown separate images of each of the eight different insects (one per trial, for a total of 8 trials), and asked to estimate the value of each insect based on its perceived likelihood of producing a golden egg.

In this experiment, we set out to test whether providing semantic labels in a first learning phase, can also help attention during reinforcement learning. To this end, we again introduced participants to a learning phase where they either learned about insects in a Semantic-labeling group or No-labeling group (e.g., similar to Experiment 1), which was now followed by a new, value-based decision-making paradigm on the same stimuli, where they needed to pay attention to all three features at the same time for optimal decision making (closely inspired by Farashahi & Soltani, 2021). Among these features, antennae thickness was partially informative about the overall insect value (Figure 5B). Insects with thick antennae had an average reward probability of 35%, while those with thin antennae had an average reward probability of 65%, indicating a higher value for insects with thin antennae. In contrast, leg thickness and mandible shape did not individually predict insect value, but factorial combinations of these features did. In other words, in the test phase we now used a value-based decision-making task, where thickness of antennae features allowed for partial generalization, but it was ultimately more beneficial to learn about the combination of features or individual objects. For example, while insects with thin antennae generally had higher values, there were exceptions where an insect with thin antennae (e.g., insect 5, Figure 5B) could have a lower value than ones with thick antennae (e.g., insect 2, Figure 5B), as illustrated in the reward probability table (see Figure 5B). Therefore, while feature-based learning might have been helpful at first, participants were ultimately prompted to adopt an object-based learning strategy.

Research has shown that well-organized representations can facilitate knowledge transfer and improve performance in related tasks (e.g., Badre et al., 2021; Bransford et al., 2000; Vaidya & Badre, 2022). Therefore, by examining the correlation between accuracy in both phases, we could assess whether access to a semantically meaningful reference frame led to enhanced task performance during the subsequent value-based decision-making task. We also investigated whether participants would adopt a feature-based or object-based learning approach in the subsequent value-based decision-making phase. Farashahi et al. (2020) compared value learning with naturalistic and abstract stimuli, showing that while both groups initially used a feature-based strategy, those exposed to naturalistic stimuli shifted more quickly to object-based learning. Although we used the same insect stimuli across both the Semantic-labeling and No-labeling conditions, we hypothesized that the availability of a semantic embedding in the Semantic-labeling group could function similarly to the naturalistic stimuli in the study by Farashahi et al. (2020), by providing a meaningful reference frame that ultimately also facilitated a transition to object-based learning. In contrast, the No-labeling condition, akin to abstract stimuli, likely lacks such a semantic framework, making it harder for participants to treat the stimuli as distinct objects, thus encouraging a feature-based learning strategy.

### Methods

#### Participants.

We recruited 191 participants (*N* = 191) via Prolific from the UK population, with the additional restriction of having an acceptance rate higher than 95%. Before starting the study, participants provided consent in accordance with the local ethics guidelines of Ghent University. Participants were removed based on the same performance criteria as Experiment 1 and 2 (i.e., a mean response time faster than 300 ms, not responding more than one-third of the trials, or pressing the same key continuously for more than 90% of trials, for any block; or an accuracy lower than 57% in the second half of the learning phase).

Our paradigm in Experiment 3 included two parts: a learning phase and a value-based decision-making task. Similar to Experiments 1 and 2, we did not apply performance accuracy criteria to the test phase, which, for Experiment 3, was the value-based decision-making phase. Performance accuracy criteria were applied only to the learning phase, as accuracy was critical to confirm initial task engagement, whereas the value-based decision-making phase was designed to capture choice patterns independent of response accuracy. From the initial 191 participants, we excluded 23.3% (45 participants) from the initial learning phase, and an additional 15.2% (29 participants) were removed from the value-based decision-making phase, totaling a 38.5% exclusion rate across both phases. While this exclusion rate for the learning phase was higher than our Prolific sample for Experiment 1 (i.e., 10.8%), this could reflect a difference in population and time (US vs. UK, and 2022 vs. 2023). Moreover, our exclusion criteria were still comparable to those of Flesch et al. (2018), who similarly examined continual learning in humans using a single paradigm and reported a 24.1% exclusion rate. Data collection continued until at least 51 participants were retained per group, yielding a final sample of 117 participants aged between 18 and 35 years. There were no significant differences in the mean age of participants across the two groups: No-labeling, with a mean age of 28.87 ± 4.56 years (31 female, 27 male); and Semantic-labeling, with a mean age of 29.2 ± 3.81 years (28 female, 30 male, 1 other).

Halfway data collection (after having tested 58 participants in the Semantic-labeling group and 59 participants in the No-labeling group), we made two minor revisions to our value-based decision-making task instructions as some participants gave feedback that they thought the second part of the paradigm was purely random (these participants were contacted because they pressed buttons faster than 300 ms, and were excluded from the analyses in accordance with our exclusion criteria). Specifically, to further encourage participants to learn in the value-based decision making task, we added the sentence: “*You will learn more about these insects through trial and error, just like in the first part.*”, and added the words “*unique*” and “*wisely*”, to the sentences “Every insect has a *unique* chance to lay magical golden eggs.” and “Choose *wisely* the one you think might have the golden touch.” to further clarify that accurate decisions could increase their chances of receiving a reward. Importantly, these minor changes were made for both experimental groups only to lower the chances of having to exclude participants from analyses, and we have no theoretical reasons to assume they could have affected our results. The exclusion rates for the value-based decision-making task remained highly similar before and after these adjustments: in the No-labeling group, we initially excluded 11 out of 59 participants (18.6%) in the first half of data collection, and in the second half, after revisions, we excluded 6 out of 39 participants (15.4%). In the Semantic-labeling group, we initially excluded 8 out of 58 participants (13.8%) in the first run and 4 out of 35 participants (11.4%) in the second run. Therefore, we included all remaining participants in one analysis.

#### Stimuli and Materials.

We used the same stimuli as Experiments 1 and 2 (Figure 5B).

#### Task and Procedure.

We only considered the Stimulus-first presentation order from Experiment 1 using Semantic-labeling vs. No-labeling conditions (Figure 5C). The procedure for the learning phase was identical to that of Experiment 1, where participants in both the Semantic-labeling and No-labeling groups performed six blocks of 48 trials each during the learning phase to learn three different tasks. The key change in this experiment was that, instead of the test phase of Experiment 1, participants proceeded directly to a value-based decision-making task—as a test of whether Semantic labeling could also benefit subsequent reinforcement learning strategies in an independent task. In this value-based decision-making task, which similarly lasted six blocks of 56 trials, participants were told to imagine they were on the fictional ‘*WonderLand*’ Planet, where their collection of insects could lay ‘golden eggs’ like hidden treasures. In each trial, they encountered pairs of insects and had to choose which insect they thought was more likely to lay a golden egg. Each insect had a unique chance to lay magical golden eggs, and the goal was to pick the insect with the best chance. Participants were reminded that sometimes none of the insects would lay golden eggs, even with careful choices. Learning happened through trial and error, similar to the learning phase of the experiment, only now participants had to learn different contingencies.

A self-paced break of up to two minutes was allowed after each block. Each trial began with a fixation cross displayed at the center of the screen for 500 milliseconds. Next, an image of a new planet appeared at the center, with a pair of insects on each side and response buttons (K & D) under the insects. Participants had 5000 milliseconds to decide which insect had a higher value by pressing the D or K buttons on the keyboard corresponding to the left or right insect. In each trial, the selection of a stimulus was rewarded according to a probabilistic reward schedule (Figure 5A). Feedback was provided with a golden egg displayed under the insect images for 1000 milliseconds. Finally, at the end of each block, a Value-Estimation task was conducted in which each stimulus was presented on the screen, and participants were asked to estimate the value of each insect on a scale from 0 to 100 (Figure 5B).

#### Reinforcement Learning Models.

We modeled the behavior of our participants during the value-based decision-making phase using reinforcement learning models designed to quantify the extent to which participants relied on feature-based versus object-based learning strategies (see also Farashahi & Soltani, 2021). Additionally, these models allowed us to estimate the weights participants assigned to different stimulus features when using a feature-based learning approach.

##### Feature-Based Models.

Feature-based models determine the reward probabilities associated with each stimulus by integrating separate feature values derived from trial feedback, akin to conventional reinforcement learning (RL) models. Specifically, the logistic function for calculating the probability *P*_*S*1_ of selecting stimulus 1 is defined as:

$$\begin{array}{l} \text{logit}\;P_{S1}\left(t\right)=W_{\text{Leg}}*\left(V_{\text{Leg}1}\left(t\right)-V_{\text{Leg}2}\left(t\right)\right)\\ \;+W_{\text{Antennae}}*\left(V_{\text{Antennae}1}\left(t\right)-V_{\text{Antennae}2}\left(t\right)\right)\\ \;+W_{\text{Mandible}}*\left(V_{\text{Mandible}1}\left(t\right)-V_{\text{Mandible}2}\left(t\right)\right)+\text{bias} \end{array}$$

*V*_*Leg*1_(*t*) and *V*_*Leg*2_(*t*) are the estimated reward probabilities associated with the leg feature of stimuli 1 and 2 respectively, and *t* denotes trial number. Similarly the *V*_*Antennae*1_(*t*) and *V*_*Antennae*2_(*t*) are associated with the antennae feature and the *V*_*Mandible*1_(*t*) and *V*_*Mandible*2_(*t*) are associated with the mandible feature. *W*_*Leg*_, *W*_*Antennae*_, and *W*_*Mandible*_ show the influence of differences in reward probabilities associated with these separate features (Leg, Mandible, Antennae) of given stimuli on choice. The bias adjusts for any bias towards left or right stimuli based on a potential location preference.

The value of each feature (leg, antennae, mandible) is updated after feedback for the chosen feature, using distinct learning rates: *α*_*reward*_ for rewarded trials and*α*_*unreward*_ for unrewarded trials. This approach, which has been shown to considerably improve model fit (Farashahi & Soltani, 2021), updates the feature values of the chosen option *V*_*chosen*_*feature*_(*t* + 1) according to the following rules:

$$\begin{array}{ll} V_{\text{chosen}\_\text{feature}}\left(t+1\right)=V_{\text{chosen}\_\text{feature}}\left(t\right)+\alpha_{\text{reward}}*\left(1-V_{\text{chosen}\_\text{feature}}\left(t\right)\right) & \text{if}\;r\left(t\right)=1.\\ V_{\text{chosen}\_\text{feature}}\left(t+1\right)=V_{\text{chosen}\_\text{feature}}\left(t\right)-\alpha_{\text{unreward}}*V_{\text{chosen}\_\text{feature}}\left(t\right) & \text{if}\;r\left(t\right)=0. \end{array}$$

##### Object-Based Models.

In object-based learning, the choices are made based on the individual insects (i.e., “objects”), and values are stored for each insect separately. Specifically, choice probabilities between two stimuli (*S*1 and *S*2) in each trial were computed according to this logistic function:

$$\text{logit}\;P_{S1}\left(t\right)=W_{o}*\left(V_{S1}\left(t\right)-V_{S2}\left(t\right)\right)+\text{bias}$$

where *P*_*S*1_(*t*) represents the probability of selecting stimulus 1, *V*_*S*1_(*t*) and *V*_*S*2_(*t*) denote the estimated reward probabilities associated with stimuli 1 and 2 respectively, *bias* measures the participant’s tendency towards left or right stimuli, and *W*_*o*_ determines the degree with which the difference in reward probabilities of the presented pair of stimuli influenced decision-making.

Object-based models directly compute the likelihood of receiving rewards for the individual stimuli (i.e., insects) based on trial feedback. Each trial, the model updates the value of the chosen stimulus using distinct learning rates for rewarded and unrewarded outcomes. The update equation for *V*_*chosen*_(*t* + 1) is:

$$\begin{array}{ll} V_{\text{chosen}}\left(t+1\right)=V_{\text{chosen}}\left(t\right)+\alpha_{\text{reward}}*\left(1-V_{\text{chosen}}\left(t\right)\right) & \text{if}\;r\left(t\right)=1.\\ V_{\text{chosen}}\left(t+1\right)=V_{\text{chosen}}\left(t\right)-\alpha_{\text{unreward}}*V_{\text{chosen}}\left(t\right) & \text{if}\;r\left(t\right)=0. \end{array}$$

where *α*_*reward*_ is the learning rate for rewarded trials, and *α*_*unreward*_ is the learning rate for unrewarded trials.

##### Using Decay on Unselected Items.

The above-mentioned models were also extended with a decay parameter to explore the impact of decay on the estimated reward probabilities for unchosen stimuli or features. In this context, decay refers to the tendency for these probabilities to move towards 0.5 over time, at a rate determined by the decay parameter, *d*. The decay is mathematically expressed as:

$$V_{\text{Unchosen}}\left(t+1\right)=V_{\text{Unchosen}}\left(t\right)-d*\left(V_{\text{Unchosen}}\left(t\right)-0.5\right)$$

where *t* represents the trial number, and *V*_*Unchosen*_(*t*) is the estimated reward probability for an unchosen stimulus or feature. Over successive trials, this process models how unselected items gradually lose their associative strength, reverting towards an uncertainty level (0.5).

##### Reinforcement Learning Models Fitting.

To fit these models to individual participants’ choices, we used the ‘differential_evolution’ function from the ‘scipy.optimize’ library in Python. This global optimization algorithm emulates evolution by natural selection to evolve the best-fitting combination of parameters, starting from a population of random combinations. It minimizes the negative log likelihood (LL) of the predicted choice probability by searching across a broad parameter space, providing a more comprehensive exploration of potential model parameters compared to local optimization methods (Storn & Price, 1997). This approach helps ensure that the model fitting process does not become trapped in suboptimal solutions (i.e. local optima), resulting in a more robust estimation of the model parameters that best capture participants’ choice behaviour. We determined the most appropriate model by evaluating model performance using two metrics: the average negative log likelihood and the Bayesian Information Criterion (*BIC*). Furthermore, we also computed the Bayesian Information Criterion per trial, denoted as *BIC*_*p*_, following the method of Farashahi et al. (2018), to assess how well each model adapted to changes in participants’ decisions over time. The formula for *BIC*_*p*_ is:

$${BIC}_{p}\left(t\right)=-2\;LL\left(t\right)+2*k*\text{log}\left(N_{\text{trials}}\right)/N_{\text{trials}}$$

where *k* denotes the number of parameters, *t* represents the trial number, *LL*(*t*) is the log likelihood at trial *t*, and *N*_*trials*_ is the total number of trials in the experiment. By normalizing the penalty terms in *BIC* by the number of trials, we ensure that the cumulative sum of *BIC*_*p*_, across all trials, is equivalent to the global *BIC*. Lower scores on these measures suggest a stronger alignment between the model and the observed behavior, thus allowing us to identify the most accurate representation of participants’ decision-making processes. This measure allowed us to investigate how feature-based versus object-based reinforcement learning strategies evolved over time.

### Results

#### Behavioral Data Analysis.

Similar to the previous experiments, we first focused on the average performance accuracy scores across the different experimental phases (learning and test phases), as well as the potential relations between them.

#### The Effect of Semantic Labeling on Task Learning.

**Figure 6.**
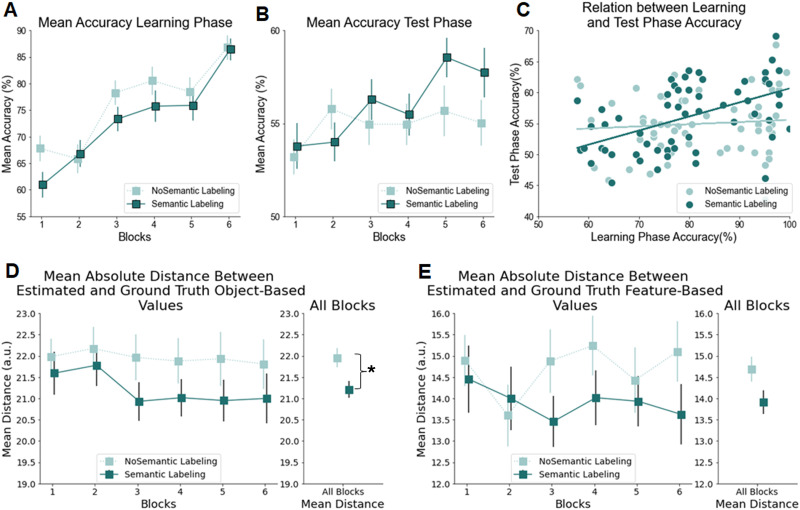
**Experiment 3 Behavioral Results.** *Note*: All error bars represent the standard error of the mean (*SEM*). **(A)** Learning Curves in the learning phase: Mean accuracy, averaged over 48 trials per block. No significant differences in accuracy were found between the groups. **(B)** Learning Curves for the value-based decision-making task: Mean accuracy, averaged over 56 trials per block. **(C)** Correlation Between Accuracy in the learning phase (i.e., continual learning task) and the value-based decision-making task: A strong positive correlation, *r* = 0.52, was observed in the semantic-labeling group, while the no-labeling group showed a weaker correlation, *r* = 0.08. A Fisher’s z-test revealed a significant difference between these two correlations, *p* = .01. **(D)** Mean Absolute Distance Between Estimated Value and Object-Based True Values: The mean absolute distance, averaged over 8 trials per block, measures how closely participants estimated the true object-based values of insects. The average across all blocks, as displayed on the right side of the plot, shows a significant difference between the groups in correctly estimating insect values, *p* = .013. **(E)** Mean Absolute Distance Between Estimated Value and Feature-Based True Values: Similar to (D), this panel presents the mean absolute distance averaged over 8 trials per block, but it focuses on how closely participants estimated insect values based on a feature-based strategy. The average across all blocks revealed no significant difference between groups, *p* = .055.

In line with the findings from Experiments 1 and 2, we found no significant differences between the Semantic-labeling and No-labeling groups during task learning, indicating that both groups learned the different tasks at comparable levels. Specifically, when comparing the mean accuracy (Blocks 4, 5, and 6) between participants in the Semantic-labeling (Stimulus-first Semantic-labeling) and No-labeling (Stimulus-first No-labeling) groups during the learning phase, the mean accuracy was M = 0.794 (SD = 0.13) for the Semantic-labeling group and M = 0.819 (SD = 0.12) for the No-labeling group (Figure 6A). A two-sample *t*-test indicated no significant difference in mean accuracy between these two groups, *t*(115) = −1.125, *p* = .263, suggesting that the true difference in means is not significantly different from zero (the 95% confidence interval for the difference in means ranged from −0.071 to 0.020).

#### Impact of Semantic Labeling on Subsequent Value-Based Decision-Making.

Next, semantic labeling in the learning phase did not appear to have major effects on choice accuracy in the value-based decision-making task, but did result in more correct value estimations. We expected that labeling in the learning phase could enhance accuracy in the value-based decision-making task. However, a first, overall ANOVA did not bring support for this. We performed a repeated measures ANOVA to examine the effects of Label (Semantic-label vs. No-label) and Time (6 blocks) on accuracy in the value-based decision-making task. The main effect of Label was not statistically significant, *F*(1, 115) = 1.14, *p* = .288, indicating no significant difference in accuracy scores between the Semantic-labeling and No-labeling groups across all time points. The main effect of Time was statistically significant, *F*(5, 575) = 3.039, *p* = .01, suggesting accuracy improved over the course of this task, indicating learning (see Figure 6B). The interaction between Time and Label was not statistically significant, *F*(5, 575) = 1.42, *p* = .215. However, to further investigate potential differences between the impact of Semantic-labeling vs. No-labeling, we also zoomed in on the most sensitive period to assess differential learning effects, and conducted a separate analysis on the last three blocks of the value-based decision-making task, where potential differential learning effects may have accumulated most. A repeated measures ANOVA showed again a non-significant effect of label, *F*(1, 115) = 2.75, *p* = .099. Although this result is not statistically significant, it suggests that an accuracy difference could become apparent with additional training. In summary, while there was no evidence of a labeling effect across all six blocks, a numerical trend of Label toward the end suggests that semantic labeling might have shown an influence on performance as participants become more familiar with the task (Figures 6A, 6B).

Semantic labeling did lead to more accurate value estimations compared to the No-labeling group, when estimating object-based insect values. After each block of the value-based decision-making task, participants completed a value-estimation task where they estimated the value of each insect on a scale from 0 to 100, indicating the probability of the insect laying a gold egg (see Figure 5B). To analyze participants’ responses in the value-estimation task, absolute distances were calculated for each insect between participants’ estimated values and the actual object-based values. A two-sample *t*-test, comparing the mean absolute distances between the Semantic-labeling group (mean = 21.21) and the No-labeling group (mean = 21.95), revealed a statistically significant difference, *t* = −2.49, *p* = .013. In other words, participants’ value estimations for each insect in the Semantic-labeling group were significantly closer to the true insect values compared to those in the No-labeling group. This suggests that participants in the Labeling group provided more accurate estimations overall, with values that were, on average, closer to the ground truth object-based values than participants in the No-labeling group (Figure 6D). To assess potential feature-based generalization, we also computed the distance between each estimated object value and the average ground truth value associated with its informative feature (i.e., antenna type: thick = 3.5, thin = 6.5). A two-sample *t*-test comparing the mean absolute distances between the Semantic-labeling group (mean = 13.92, SE = 0.28) and the No-labeling group (mean = 14.69, SE = 0.29) was not significant, *t* = −1.93, *p* = .055.

#### Correlations Between Both Forms of Learning Across Phases.

Next, a correlation analysis demonstrated that participants in the Semantic-labeling group showed a stronger relation in performance between the learning phase of the continual learning task and the value-based decision-making task, compared to the No-Labeling group. As discussed in the introduction, we aimed to investigate the potential benefit of building more separate task representations in the Semantic labeling group on performance in the value-based decision-making task, by examining the correlation between their respective accuracy scores. Pearson correlation coefficients were computed between the mean accuracy of Blocks 4, 5, and 6 from the initial learning phase and the overall mean accuracy on the value-based decision-making task. The correlation coefficient for the Semantic-labeling group was *r* = 0.52, *p* < .001, indicating a strong positive relationship between accuracy scores across the two phases. In contrast, the No-Labeling group exhibited a much weaker correlation of *r* = 0.08, *p* = .527, suggesting a lack of association between both tasks. To determine if these correlation coefficients differed significantly between the Semantic-labeling and No-labeling groups, Fisher’s *z* transformation method was conducted, which revealed a statistically significant difference between both correlation coefficients, Fisher’s *z* = 2.578, *p* = .01 (Figure 6C). Overall, these results suggest that semantic labeling not only consolidates task knowledge in the learned task but also fosters learning on other tasks, potentially by providing a more structured reference frame for learning about the different insect features.

To ensure a comprehensive picture of the results, we also conducted these correlation analyses for Experiments 1 and 2, examining the relationship between learning-phase (Blocks 4–6) and test-phase performance (Blocks 7–9). Importantly, the reasoning is different for these analyses as they do not evaluate correlations between the learning of two different tasks, but whether learned task knowledge in the training phase correlated with that same task knowledge in the test phase. In Experiment 1, we observed a clear correlation in both the Labeling condition (*r* = 0.91, *p* < .001) and the No-labeling condition (*r* = 0.76, *p* < .001), which significantly differed from each other (*Z* = 4.48, *p* < .001). In Experiment 2, both the Symbol-label and Word-label conditions showed strong correlations between learning and test performance (*r* = 0.80 and 0.86, respectively; both *p* < .001), with no significant difference between them (*Z* = –0.669, *p* > .05).

#### The Effect of Semantic Labeling on Different Reinforcement Learning Strategies.

Finally, we used reinforcement learning modelling to study how semantic labeling influenced learning and attention during the value-based decision-making task, by comparing feature-based and object-based reinforcement learning approaches, as well as how participants assigned attentional weights to each feature. We fit human data to four different RL models and calculated the Bayesian Information Criterion (BIC) as a measure of goodness-of-fit. Our analysis included comparisons of BIC values across groups, the weights associated with each feature, and an investigation into the potential transition between learning strategies throughout the training phase.

The feature-based decay model outperformed the object-based decay model in both groups, and both models fit better in the Semantic Labeling group. Overall, we found smaller mean BIC values (lower values indicate a better fit) in the Labeling condition, representing a better fit for all models compared to the No-labeling condition. The models that incorporated a decay in value estimates over time fitted best in both Labeling and No-labeling conditions. Contrary to our expectation that the Semantic-labeling group would predominantly use an object-based learning strategy, we found that the feature-based model with decay provided the best fit for both groups (Table 1). Specifically, the feature-based with decay model exhibited a significantly superior fit compared to the object-based with decay model in both the Semantic-Labeling and No-labeling conditions. For the No-labeling group, the mean difference in BIC values (*BIC*_feature−based with decay_ − *BIC*_object−based with decay_) was 5.51 ± 2.83, *p* = .024, *d* = 0.26, indicating a statistically significant but small effect. In contrast, for the Semantic-label group, the mean difference in *BIC* values (*BIC*_feature−based with decay_ − *BIC*_object−based with decay_) was 13.39 ± 2.81, *p* < .001, *d* = 0.62, demonstrating a significant and moderately large effect. We used a Mann-Whitney *U* test to compare the BIC differences (*BIC*_feature−based with decay_ − *BIC*_object−based with decay_) between the Labeling and No-labeling groups. The results showed no statistically significant difference (*p* = .107). Finally, we also conducted direct comparisons between the groups for both the object-based and feature-based models. For both the object-based and feature-based models, there was a statistically significant difference between the Labeling and No-labeling groups, Mann-Whitney *U* = 2102.00, *p* = .033, and Mann-Whitney *U* = 2110.00, *p* = .029, respectively. Together, these findings suggest that while both groups favored the feature-based decay model, the presence of semantic labels during initial task learning did result in subsequent improved participants’ learning strategies, leading to significant improvements in how object-based and feature-based models fit performance data in the Semantic-Labeling versus No-Labeling group.

**Table 1.** Comparison of goodness-of-fit metrics for reinforcement learning models on the value-based decision-making task, based on prior access to Semantic-labeling versus No-labeling during previous task learning.

	***Model***	***BIC***	***NegLL***	***Number of Parameters***
**No-Label**	Object-Based	461.649 ± 4.23	219.197 ± 2.11	4
	Object-Based Decay	455.327 ± 5.33	213.129 ± 2.66	5
	Feature-Based	451.687 ± 6.40	208.402 ± 3.20	6
	Feature-Based Decay	449.820 ± 6.81	204.562 ± 3.40	7
**Semantic-Label**	Object-Based	447.328 ± 5.43	212.035 ± 2.71	4
	Object-Based Decay	438.488 ± 6.53	204.707 ± 3.26	5
	Feature-Based	429.269 ± 8.08	197.190 ± 4.04	6
	Feature-Based Decay	425.101 ± 8.651	192.199 ± 4.32	7

*Note*: Two goodness-of-fit metrics—negative log likelihood (−LL) and Bayesian information criterion (BIC)—were calculated and averaged over participants (mean ± s.e.m.) for both feature-based RL models and their object-based counterparts. Lower values indicate a better model fit. The feature-based model with decay provided the best fit for the choice data across both groups. Specifically, the feature-based with decay model showed a significantly superior fit compared to the object-based with decay model in both the Semantic-Labeling and No-labeling conditions. Feature-based with decay RL models were compared to their object-based counterparts using a two-sided signed-rank test.

**Figure 7.**
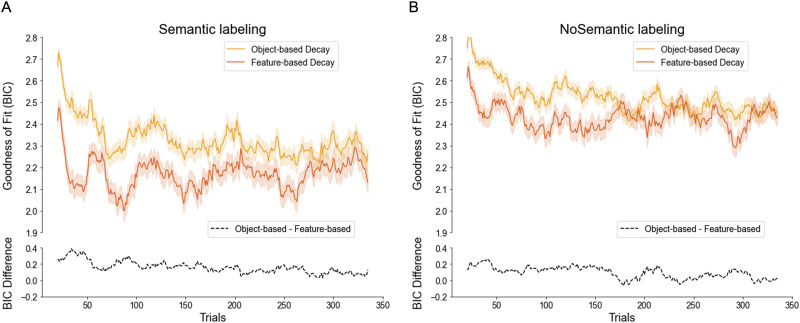
**Time course of BIC per trial for object-based and feature-based models with decay during the experiment.** *Note*: (A, B) Shown is the goodness-of-fit, represented by the average BIC per trial (*BIC*_*p*_), for both models with decay in the Semantic-labeling and No-labeling groups. Lower values indicate a better fit. Shaded areas represent mean ± s.e.m. The running average is calculated using a moving window of 20 trials. The dashed black curve depicts the difference in goodness-of-fit between the object-based and feature-based models with decay (*BIC*_*object*−*based with decay*_ − *BIC*_*feature*−*based with decay*_) in the Semantic-labeling and No-labeling groups.

By analyzing participants’ choice data and calculating the BIC for each trial (*BIC*_*p*_; see Methods section), as different measures of goodness-of-fit (see Farashahi et al., 2018), we also aimed to determine whether participants in the Semantic-labeling condition transitioned to an object-based strategy earlier than those in the No-labeling condition. We hypothesized that participants provided with semantic labels would show a faster shift toward an object-based learning strategy, reflected by a more negative slope in the difference between the BIC values of object-based and feature-based models over time (Figure 7). However, statistical analysis of the BIC slopes did not support this hypothesis. An independent *t*-test comparing the slopes between the two groups revealed no significant difference between the Semantic-labeling and No-labeling groups, *t* = −0.2367, *p* = .813. This result suggests that the rate of change in the relative goodness-of-fit between the object-based and feature-based models was comparable across both conditions, indicating no earlier transition to an object-based strategy in the Semantic-labeling group (Figures 7A, B). Additionally, we examined whether the slope of each group was significantly different from zero, as an indicator of the more general, hypothesized shift toward an object-based strategy over time. The mean slope in the Semantic-labeling (SBL) group was significantly different from zero, *t* = −2.789, *p* = .007, as was the mean slope in the No-labeling (SBP) group, *t* = −2.496, *p* = .015. These results provide evidence for an overall negative trend in both groups, suggesting that participants in both conditions showed a gradual shift toward an object-based learning strategy.

Finally, we studied the separate parameters in the feature-based model, identified as the best fitting model for both groups, to investigate potential differences in attentional weights to the three different features between the Semantic-Labeling versus No-Labeling groups. To further investigate the specific contributions of the parameters in the feature-based decay model, we focused on the weights assigned to each stimulus feature: leg thickness, antennae thickness, and mandible shape. These weights, denoted as *W*_*f*_ = {*W*_*Leg*_, *W*_*Antennae*_, and *W*_*Mandible*_} represent the cognitive weights or selective attention during task performance. A larger value of *W*_*f*_ indicates a greater emphasis placed on that particular feature during decision-making.

By comparing these parameters across groups, we aimed to understand how access to semantic labeling during the initial continual task learning phase impacted the assignment of weights to each feature during subsequent reinforcement learning. A mixed 2 × 3 ANOVA was conducted on the Log *W*_*f*_ values (log-transformed to normalize the distribution), with Label (Semantic-label vs. No-label) as the between-subjects factor and Feature (Antennae vs. Mandible vs. Leg) as the within-subjects factor. The main effect of Feature was not significant, *F*(2, 345) = 2.607, *p* = .075. The main effect of Label was also not significant *F*(1, 345) = 1.270, *p* = .26.

**Figure 8.**
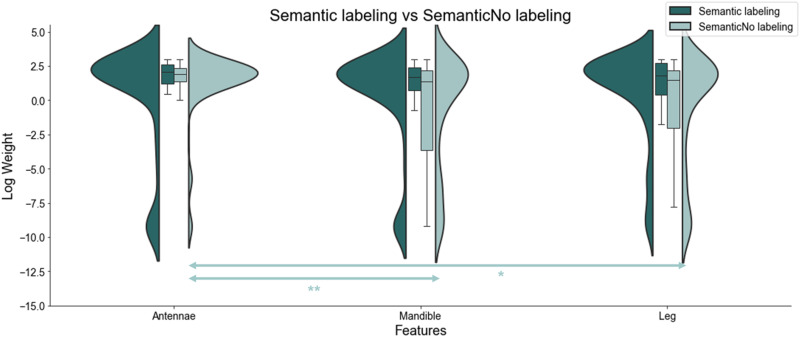
**Distribution of the log-transformed feature weights (Log *W*_*f*_) used by the feature-based with decay reinforcement learning (RL) model to make decisions based on different stimulus features.** *Note*: In the Semantic-label group, there was no significant difference between the three log *W*_*f*_, but in No-label group the log *W*_*Antennae*_, was significantly different from *W*_*Leg*_ and *W*_*Mandible*_.

However, there was a significant interaction between Feature and Label, *F*(2, 345) = 3.06, *p* = .048, indicating that the influence of feature type on weighting depended on the labeling condition. To unpack this interaction effect, we performed separate ANOVAs for each group (Semantic-label vs. No-Label), followed by post-hoc pairwise comparisons with Bonferroni correction. The one-way ANOVA in the Semantic-label group did not show a significant effect of feature type on Log *W*_*f*_ values (*F*(2, 174) = 0.052, *p* =.949). This suggests that participants in the Semantic-labeling group assigned similar weights to all features (Antennae, Leg, and Mandible; Figure 8A). In contrast, the one-way ANOVA for the No-Label condition revealed a significant effect of feature type on Log *W*_*f*_ values (*F*(2, 171) = 5.621, *p* = .004). This indicates that participants in the No-Label group assigned different weights to the features. Pairwise *t*-tests with Bonferroni correction were conducted to determine which features differed significantly in the No-Label group. The results indicated that Log *W*_*f*_ values for Antennae were significantly different from both Leg (*p.adj* = .017) and Mandible (*p.adj* = .009). However, there was no significant difference between Log *W*_*f*_ values for Leg and Mandible (*p.adj* = 1.00; Figure 8B). These findings suggest that participants in the Labeling group allocated equal weight to all three features (Antennae, Leg, and Mandible) during decision-making. Conversely, participants in the No-Label condition paid significantly more attention to the partially informative feature (Antennae) while making their decisions.

### Discussion

Our third experiment provides insights into how semantic labeling can impact future reinforcement learning strategies. We showed that access to a semantic task embedding during continual learning had an impact on learning about new reward contingencies in a subsequent value-based decision-making task. Namely, we demonstrate how performance between both phases was related, only when a semantically rich task embedding was provided in the first phase. Moreover, learning about the stimuli under semantic labeling conditions resulted in better performance in the value estimation tasks when asked about the learned reward values. This suggests that semantic anchors may have lasting effects and help improve performance across tasks. We also demonstrated that semantic labeling influences reinforcement learning strategies. In particular, in the feature-based model, which emerged as the best-fitting model for both groups, we observed that participants in the Semantic-labeling group showed a more uniform distribution of cognitive weights across the three features, indicating a more balanced consideration of each feature during decision-making. In contrast, participants in the No-Label group placed significantly greater emphasis on the only feature across which they could generalize. These findings align with our results from the value estimation task, where we observed significant differences between the groups in estimating the value of stimuli using an object-based approach. This further supports the idea that semantic labeling enhances accurate value estimation by promoting a more balanced consideration of all relevant features. In summary, Experiment 3 underscores the critical role of semantic labeling in facilitating attentional weighting and influencing reinforcement learning strategies, demonstrating how semantic anchors can enhance cognitive flexibility and improve task performance.

## EXPERIMENT 4

In this fourth experiment, we wanted to determine whether the observed benefits of our Semantic Labeling manipulation were truly due to the semantic richness in which tasks could be embedded, or whether it was due to the use of task-specific response labels. Namely, in our Semantic Labeling condition, participants chose between conceptually meaningful alternatives (e.g., *Warm* vs. *Cold*, *High* vs. *Low*), which we assumed supported learning by leveraging a pre-existing semantic structure in which the newly learned tasks could be embedded. In both Experiment 1 and 3, we contrasted this with a No-labeling condition using binary decisions about whether an insect could live on a given fictional planet (e.g., *Yes* or *No*), with planet identities indicated by color (e.g., *pink, blue*), offering no inherent semantic structure—as people typically do not use planets to categorize animal species. However, while both groups made two-alternative choices, the No-labeling condition may have introduced more overlap across tasks due to the abstract and interchangeable nature of the response labels (e.g., the same *Yes*/*No* responses applied to different planet colors). This could have increased interference and reduced learning performance, independent of the semantic content. To test this, we directly compared the original semantic-labeling condition with a new control condition: the shape-labeling condition. In this shape-labeling group, participants again made two-alternative choices, but using arbitrary visual symbols (e.g., *triangle* vs. *star*) that, unlike the semantic labels, lacked any conceptual meaning. If semantic content is the key factor supporting learning and reducing interference, we again expected stronger task performance and retaining of task information in the semantic-labeling condition as opposed to the shape-labeling condition.

### Methods

#### Participants.

We recruited 249 first-year psychology students from Ghent University via the SONA system who performed the experiment in exchange for course credit. Exclusion criteria matched those in Experiment 1: Participants were again removed for mean RTs below 300 ms, missing over one-third of trials, or repeating the same key press in over 90% of trials within a block. Based on these criteria, 7 participants were excluded from the Shape-labeling group and three from the Semantic-labeling group. An additional 10 (Shape) and 18 (Semantic) were excluded for scoring below 57% accuracy in the second half of the learning phase. The final sample included 108 participants in the Shape-labeling group (Mean age = 18.86 ± 2.4; 97 female, 10 male, two other) and 105 in the Semantic-labeling group (Mean age = 18.92 ± 3.1; 92 female, 11 male, two other). We used a larger sample in Experiment 4 to ensure adequate power for detecting potentially smaller effects, given the subtler contrast between two labeling conditions—semantic versus shape—both of which may provide some structural support by using different response labels.

#### Task and Procedure.

**Figure 9.**
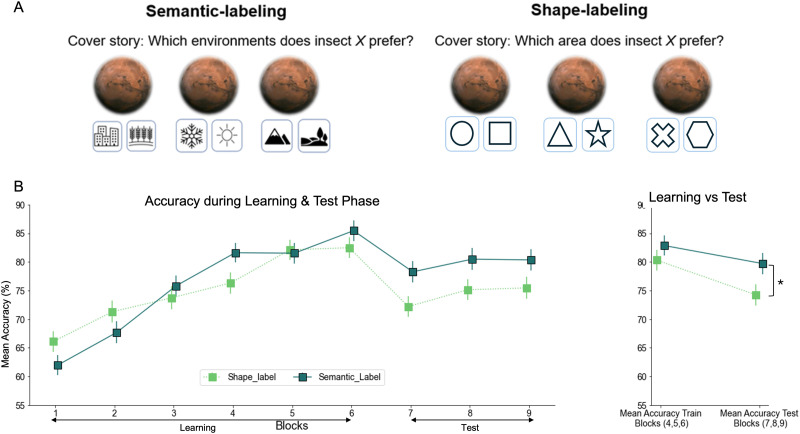
**Experiment 4: Task Design and Results.** *Note*: Error bars represent the standard error of the mean (*SEM*). **(A)** Response options in the Semantic-label group conveyed conceptually meaningful structure (e.g., *Warm* vs. *Cold*), while the Shape-label group used arbitrary shape pairs (e.g., triangle vs. star), lacking inherent semantic association. **(B)** Mean accuracy during the late learning phase (Blocks 4–6) was compared to mean accuracy in the test phase (Blocks 7–9). While both groups showed a significant decline from learning to test (*p* < .001), overall accuracy was higher in the Semantic-labeling group than the Shape-labeling group (*p* = .040). A planned comparison confirmed that this difference was driven by significantly better performance in the Semantic-labeling group during the test phase (*p* = .03).

The overall task structure in Experiment 4 was identical to the Stimulus-first Semantic-labeling condition in Experiment 1, including the same learning and test phase design, timing, and feedback schedule (see Experiment 1: Task and Procedure). As in Experiment 1, participants in the *Semantic-labeling* group played the role of space biologists, identifying which environmental condition the insect on screen preferred (e.g., Warm vs. Cold, Rural vs. Urban, High vs. Low altitude). However, participants in the *Shape-labeling* group were given a new cover story. They were again cast as space biologists, this time tasked with identifying the insect’s preferred egg-laying environment. Instead of meaningful semantic response labels, participants chose between arbitrary shape pairs (e.g., triangle vs. star, circle vs. square, cross vs. hexagon), carefully selected to avoid any semantic associations (see Figure 9A).

### Results

#### Semantic Content—Not Response Labeling—Drives the Labeling Advantage.

##### Providing Semantic Labels, Rather Than Arbitrary Ones, Supports Task Performance Under Interference-Prone Conditions, Demonstrating That the Labeling Benefit Stems From Semantic Content, Not Response Labeling.

Similar to the previous experiment, we conducted a repeated-measures ANOVA with Time (Learning: Blocks 4–6 vs. Test: Blocks 7–9) as a within-subject factor and Group (Semantic-label vs. Shape-label) as a between-subject factor. The analysis revealed a significant main effect of Group, *F*(1, 211) = 4.29, *p* = .040, ges = .017, indicating that overall accuracy differed between the Semantic-label and Shape-label groups. There was also a significant main effect of Time, *F*(1, 211) = 27.42, *p* < .001, ges = .022, reflecting a general decline in performance from the learning to the test phase. The Group × Time interaction was not significant when tested two-sided, *F*(1, 211) = 2.69, *p* = .103, but close to significance when tested one-sided, *p* = .052 (in line with our directional hypothesis), suggesting that semantically meaningful labels helped both learning as well as reduce the decline in performance during the test phase, when retrieval demands and task interference are greatest.

### Discussion

Experiment 4 confirmed that the benefits of semantic labeling stem from conceptual content, not task structure or response format, which drives the labeling advantage. Despite identical procedures and response mappings, only semantic labels supported stable task representations and protected against forgetting and interference, strengthening the conclusion from Experiments 1 and 3.

## EXPLORATORY MODELING OF TASK LEARNING USING ARTIFICIAL NEURAL NETWORKS

In this last section, we examined whether we could use recurrent neural networks (RNNs), trained on participants’ data from Experiments 1, 3, and 4, to detect differences in task representations. Namely, we trained RNNs on the exact trial sequences and their responses as experienced by participants, allowing us to test whether manipulations—such as stimulus–context order (stimulus-first vs. context-first, Experiment 1) and labeling condition (semantic vs. no label in Experiments 1 and 3; semantic vs. shape label in Experiment 4)—produced distinguishable patterns in the networks’ internal representations, despite similar levels of accuracy during training.

By analyzing hidden layer dynamics (see Ji-An et al., 2024; Verbeke & Verguts, 2025), we aimed to explore how these different training conditions might have differentially affected the underlying representational geometry. While not directly analogous to neural data from methods like fMRI, we reasoned this approach could serve as an exploratory complement to our behavioral results, providing an alternative summary statistic of choice behavior that offered new insights above and beyond average task accuracy—keeping in mind that training accuracy was never significantly different across groups (including in Experiment 4: *t* = 1.58, *p* = .11, 95% CI [–0.006, 0.057]). This way, our goal was not to model human cognition mechanistically, but to use RNNs as descriptive tools for probing behaviorally grounded task representations.

Importantly, we hypothesized that providing a semantic embedding space would result in greater task separation to promote efficient task learning task learning. Specifically, we assumed that a larger separation between task-specific activation patterns—as quantified as the distance in hidden layer space—would correspond to a higher test-phase accuracy in the Semantic labeling group, reflecting reduced interference.

### Methods

#### Neural Network Analyses.

We used a Simple Recurrent Neural Network (SimpleRNN) with two input layers: one for the stimulus and one for the context (task-cue). These input layers fed into a single recurrent layer, followed by a dense output layer. The stimulus input consisted of 5 nodes, and the context input used 3 nodes and one-hot encoding to represent one of three different task contexts. The simplicity of the context input—using one-hot encoding without any visual features—was an intentional design choice. This ensured that any representational differences in the network’s hidden layers would not arise from the model architecture or input format, but rather from the abstract structure embedded in the participants’ behavioral responses. The recurrent layer included a total of 48 hidden neurons, utilized 10 timesteps within a trial, and was reset in between trials. Note that we explored different architectures by using a different number of hidden neurons (8, 24, 48), number of timesteps (2, 10), and reset in between trials (yes or no). However, across all groups, this architecture provided the best fit, as determined based on the lowest average test loss across groups, indicating it minimized the discrepancy between predicted and actual outcomes best on unseen data. The output layer was a dense layer with two units and SoftMax activation, designed for binary classification. For each of the four groups separately, we fitted all participants’ data from the learning phase (6 blocks of 48 trials, using participants’ responses as output instead of the nominally correct response). To simulate the effect of context-first and stimulus-first conditions, the first five timesteps of each trial only included the context or stimulus, depending on the group. The dataset was divided into a training and validation dataset. The training dataset consisted of the first 36 trials of each block, while the validation dataset comprised the last 12 trials of each block. Behavioural responses from participants were provided to the model as labels for both the training and validation datasets. We allowed the model to adjust all its weights. The network was trained using a categorical cross-entropy loss function and the Adam optimizer with a learning rate of .0001. Early stopping was employed to prevent overfitting, monitoring the validation loss with a patience of 3 epochs. The network was trained for a maximum of 10,000 epochs, with a batch size of 72.

#### Data Analysis.

Recurrent Neural Network (RNN) simulations were used to investigate how task representations evolve under two key experimental manipulations: (1) the order of stimulus and context presentation (Experiment 1), and (2) the presence versus absence of semantic labeling (Experiments 1, 3, and 4).

As an initial validation step, we assessed the model fit of the RNN models across conditions in each experiment by looking at the model’s predictive accuracy. Importantly, this accuracy refers to the model’s ability to predict participants’ responses, not task correctness, allowing us to ensure that the models learned to simulate human-like behavior comparably across all conditions. This step was critical to rule out differences in model performance as a confounding factor when analyzing task representations.

Next, we examined how task representations were encoded in the RNNs’ hidden layers. Specifically, we used the activations from this single recurrent hidden layer for the representational distance analysis to assess how the *Task Separation* score varied across experimental manipulations. To compute this score, we focused on hidden layer activations. We calculated the average Euclidean distance between representations for each of the three task comparisons for every insect (e.g., task A vs. B, B vs. C, and A vs. C), then averaged these distances across insects.

We also contrasted this measure with a *Task Specialization* score, which quantified the alignment of internal representations with normative task structures: We compared the representational dissimilarity matrices of each task (i.e., an eight by eight matric comparing the activation pattern of each insect to each other) to the respective theoretical ground-truth dissimilarity matrices using Spearman correlations (Kriegeskorte et al., 2008). The average of these three correlations yielded a *Task Specialization score*, indicating how well the model captured context-specific task structure.

To further evaluate whether either task separation or task specialization drove learning more, we used multiple regression models investigating if *Task Separation* and *Task Specialization* could predict participant’s accuracy during the training phase. In a first multiple regression model we only included the main effects for Task Separation, Task Specialization, and labeling condition (labeled vs. unlabeled).

**Model 1:**

$$\text{Mean}\_\text{Training}\_\text{Accuracy}∼\beta_{0}+\beta_{1}\text{TaskSeparation}+\beta_{2}\text{TaskSpecialization}+\beta_{3}\text{LabelingCondition}+\varepsilon .$$

Critically, in a second model we also added the interaction terms to examine whether the effects of different effects of Task separation versus Task specialization on learning performance varied as a function of labeling. All models were fitted using ordinary least squares. Continuous predictors were mean-centered prior to analysis.

**Model 2:**

$$\begin{array}{l} \text{Mean}\_\text{Training}\_\text{Accuracy}∼\beta_{0}+\beta_{1}\text{TaskSeparation}+\beta_{2}\text{TaskSpecialization}\\ \;+\beta_{3}\text{LabelingCondition}+\beta_{4}\left(\text{TaskSeparation}\times \text{LabelingCondition}\right)\\ \;+\beta_{5}\left(\text{TaskSpecialization}\times \text{LabelingCondition}\right)+\varepsilon \end{array}$$

Finally, we applied the same multiple regression model to explore whether RNN-derived representational metrics also predicted participants’ performance during the test phase.

### Results

#### RNN Fit.

**Figure 10.**
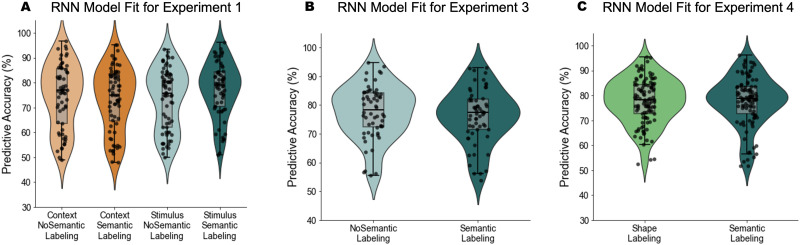
**RNN Model fits.** *Note*: RNN model accuracy did not significantly differ between groups in any experiment. **(A)** Experiment 1: No effects of labeling or presentation order. **(B)** Experiment 3: No difference between Semantic-labeling and No-labeling groups. **(C)** Experiment 4: No difference between Semantic and Shape Labeling conditions.

Across all experiments, RNN models predicted participants’ responses with comparable accuracy across conditions. In *Experiment 1*, a two-way ANOVA revealed no significant effects of Labeling (*F*(1, 261) = 0.723, *p* = .396), Order (*F*(1, 261) = 0.703, *p* = .402), or their interaction (*F*(1, 261) = 2.745, *p* = .099), indicating stable performance across conditions (Figure 10A). In *Experiment 3*, the *t*-test showed no significant accuracy difference between Semantic-labeling and No-labeling groups, *t* = −0.756, *p* = .45 (Figure 10B). Similarly, in *Experiment 4*, the *t*-test showed no difference between Semantic and Shape Labeling conditions, *t* = −0.051, *p* = .960 (Figure 10C). These results confirm that the RNNs showed similar levels of model fit across experimental manipulations, supporting the validity of subsequent representational dissimilarity analyses.

#### Task Representation Geometry Results.

**Figure 11.**
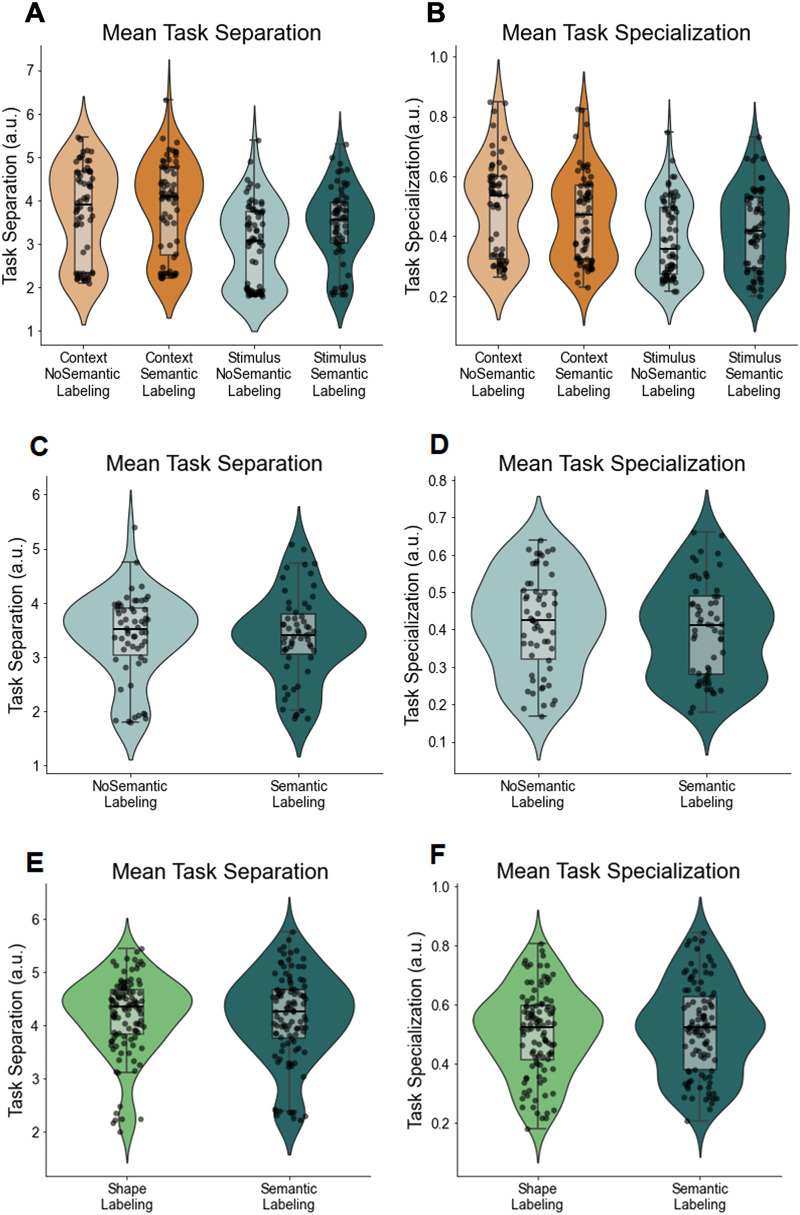
**The comparisons of representational geometry across experiments.** *Note*: Task separation and specialization scores across experiments. **(A, B)** Effects of labeling (semantic vs. no-semantic) and presentation order (context-first vs. stimulus-first) in Experiment 1. **(C, D)** Semantic-labeling vs. no-labeling in Experiment 3. **(E, F)** Semantic vs. shape-labeling in Experiment 4.

In Experiment 1, a two-way ANOVA revealed significant main effects of both labeling (*F*(1, 261) = 4.88, *p* = .028, *ges* = .02) and presentation order (*F*(1, 261) = 23.10, *p* < .001, *ges* = .08) on task separation, with higher separation scores observed in the semantic-labeling and context-first conditions (Figure 11A). For task specialization, only the main effect of order was significant (*F*(1, 261) = 16.07, *p* < .001, *ges* = .06), indicating greater specialization in the context-first condition. The main effect of labeling and the interaction were not significant (*p*s > .05; Figure 11B).

In Experiment 3, task separation did not differ between the semantic-labeling and no-labeling groups, *t* = 0.008, *p* = .994 (Figure 11C). Task specialization also showed no significant group difference, *t* = −0.70, *p* = .488 (Figure 11D).

Finally, in Experiment 4, no differences were found between the semantic- and shape-labeling groups for either metric. Task separation was comparable across groups, *t* = −0.34, *p* = .736, as was task specialization, *t* = 0.63, *p* = .528 (Figures 11E, 11F).

#### Predicting Training Accuracy From Representational Geometry.

The group level differences showed that only in Experiment 1, labeling resulted in more task separation, but not task specialization, a result that was not replicated in Experiment 3 or 4. Therefore, we next assessed whether both representational metrics—*Task Separation* and *Task Specialization*—would differentially predict task accuracy during the training phase using multiple regression analyses.

In *Experiment 1*, results showed that both Task Separation (*β* = 0.089, *p* < .001) and Task Specialization (*β* = 0.041, *p* < .001) significantly predicted training accuracy, while a model that included interaction terms did not improve model fit (Δ*R*^2^ = .003; *F*(2, 259) = 1.13, *p* = .326).

**Figure 12.**
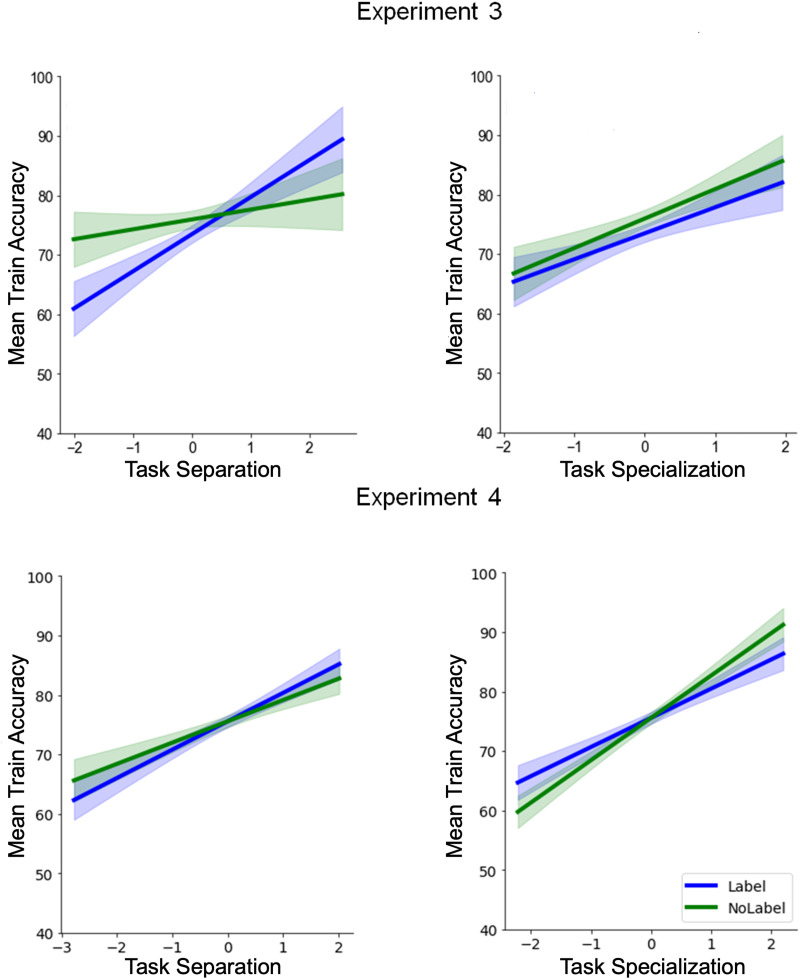
**Influence of Labeling Condition on Task Representation Geometry Predicting Training Accuracy.** *Note*: Relationship between labeling condition and task representation metrics derived from RNN hidden layers predicting training accuracy in Experiments 3 and 4.

In *Experiment 3*, both Task Separation (*β* = 0.041, *p* < .001) and Task Specialization (*β* = 0.045, *p* < .001) again predicted task accuracy, but now adding interaction terms significantly improved model fit (*F*(2, 111) = 7.76, *p* = .001). As can be seen in Figure 12A, Task Separation explained task accuracy relatively better in the Labeling as opposed to the No-Labeling group, relative to Task Specialization.

In *Experiment 4*, both Task Separation (*β* = 0.041, *p* < .001) and Task Specialization (*β* = 0.060, *p* < .001) again significantly predicted training accuracy (*R*^2^ = .746), and adding the interaction terms again improved model fit (*F*(2, 207) = 3.39, *p* = .036). Similar to Experiment 3, Task Separation again explained task accuracy relatively better in the Labeling as opposed to the No-Labeling group, relative to Task Specialization (Figure 12B).

#### Predicting Test Phase Accuracy From Representational Geometry.

Finally, we also explored whether Task Separation and Task Specialization could have helped predict participants’ performance during the test phase using the more comprehensive model with interaction terms. In both Experiments 1 and 4, which tested the effects of labeling on retaining task knowledge during the test phase, results showed that both *Task Separation* (*β* = 0.077, *p* < .001; *β* = 0.057, *p* = .001) and *Task Specialization* (*β* = 0.061, *p* < .001; *β* = 0.056, *p* = .002) significantly predicted test accuracy. However, neither interaction between labeling and Task Separation (*β* = –0.0044, *p* = .861; *β* = –0.009, *p* = .724) nor labeling and Task Specialization (*β* = 0.0026, *p* = .916; *β* = 0.017, *p* = .511) was significant.

In *Experiment 3*, we could again evaluate whether we observed an effect not on the retaining of task knowledge, but on the task learning of a separate task. Interestingly, *Task Separation* seemed to significantly predict learning in the value-based decision-making task (*β* = 0.020, *p* = .034), and this relationship was *moderated by labeling* (interaction: *β* = –0.029, *p* = .037). This interaction showed that *Task Separation* had a stronger effect on learning during the value-based decision-making task in the Labeling group (*r* = 0.38, *p* = .003) than the No-Semantic Labeling group (*r* = 0.17, *p* = .192). In contrast, *Task Specialization* did not significantly predict accuracy (*β* = 0.001, *p* = .943), nor did its interaction with labeling (*β* = 0.022, *p* = .108).

### Discussion

We conducted the RNN analysis as a complementary approach to our behavioral data to understand how the geometry of task representations influences generalization performance during the test phase. While behavioral results consistently showed no significant difference in mean task accuracy during training between the semantic labeling and no-labeling, the RNN models suggested some differences could be observed, consistent with ideas of the impact of semantic embedding on task separation. For example, in Experiment 1, we observed a greater separation between task representations in the Semantic Labeling group. No significant differences were found between groups in Task Specialization scores for labeling effects.

Moreover, our linear regression analyses showed that both RNN-derived Task Separation and Task Specialization metrics significantly predicted training accuracy. However, Task Separation further emerged as a stronger predictor for the Labeling group versus No-Labeling group, relative to the Task Specialization metric, in both Experiments 3 and 4. This pattern highlights how different aspects of representational geometry may have supported learning depending on labeling context.

These findings are particularly meaningful because the RNNs were trained directly on human behavioral data, indicating that stable accuracy—both in humans and RNNs—can arise from varied internal representations shaped by training structure (e.g., labeling and stimulus order). However, the analyses were explorative, and the results were not always replicated. Moreover, our RNN architecture and distance metrics may not fully capture representational changes induced by the semantic labeling in particular. Nonetheless, we believe our analyses could inspire future research into alternative network architectures or analytic techniques to more precisely elucidate how semantic information shapes internal representations during task learning.

## GENERAL DISCUSSION

Humans possess a remarkable capacity for lifelong learning, allowing them to acquire and retain new tasks across a wide range of domains with minimal interference (Dehaene, 2020). This ability to continuously integrate new information while preserving prior knowledge is something that artificial intelligence (AI) systems currently struggle to replicate, often facing the challenge of catastrophic forgetting (Kudithipudi et al., 2022). Investigating the mechanisms that underlie human continual learning can provide valuable insights into human cognitive processes and may even inform the development of more robust AI algorithms (Flesch et al., 2018; Kudithipudi et al., 2022). In this study, we examined how semantically informed task labels and the sequence of stimulus and task context (task cue) presentation influenced task learning.

In our first experiment, continual learning of three tasks was compared by contrasting to semantically informative versus non-semantic task (response) labels and the order of stimulus presentation—either before or after the task cue (context), in four different groups. Participants who had access to semantic labels during training maintained their accuracy from training to test—a finding that was replicated in Experiment 2, while those without such labels experienced significant drops in accuracy, especially in the stimulus-first condition, suggesting a larger vulnerability of task representation to interference without semantic support. Importantly, the results of Experiment 4 further confirmed that the benefit of semantic labeling was not due to differences in response labeling per se. Our explorative neural network model simulations were further suggestive of the idea that presenting semantically informative response labels may have enhanced task representation separability.

Our findings can be understood through the lens of language’s influence on perceptual and cognitive processes, much like other sensory inputs (Kray et al., 2004; Lupyan, 2016; Radulescu et al., 2022). Language has been shown to play a pivotal role in increasing categorical clarity, reducing ambiguity, and organizing knowledge, which in turn shapes perception, concept formation, and task representations (Davis, & Yee, 2021; Dove et al., 2022; Henningsen-Schomers et al., 2023; Lupyan, 2012b; Mikolov et al., 2018). Lupyan and Thompson-Schill (2012) demonstrated that verbal labels activate familiar knowledge in a manner that differs significantly from nonverbal cues. They explored how auditory input, such as hearing the name of an object (e.g., “dog” or “motorcycle”), influences visual recognition compared to listening to the sounds associated with those objects (e.g., a dog barking or a motorcycle engine). Their results indicated that verbal labels facilitate more effective engagement with conceptual representations, operating in a fundamentally different way than nonverbal stimuli. Specifically, the representation triggered by verbal labeling is more abstract and prototypical in nature (Edmiston & Lupyan, 2015). This body of research suggests that labeling does more than merely identify tasks; it fundamentally alters how those tasks are represented cognitively.

Our results suggesting enhanced task performance during the test phase in the semantic labeling group also provide experimental support for a recent theoretical framework proposed by Giallanza et al. (2024), which presents a unified model of semantics and cognitive control. Their model emphasizes the interplay between semantic cognition and cognitive control, suggesting that the ability to flexibly access and utilize semantic knowledge arises from learning the statistical patterns of the environment. Overall, in the context of our experiment, semantic labeling likely helped participants form more distinct and separable task representations (which our neural network simulations also partially hinted at), which have been argued to reduce task interference and forgetting (Musslick & Cohen, 2021). These abstracted, categorical representations may have allowed for quicker retrieval and better task differentiation, particularly in the more challenging stimulus-first condition, where risks of task interference were higher.

The benefits of semantic labeling observed in Experiment 1 extend beyond reducing task interference and retaining task knowledge. In Experiment 3, we found that this semantic embedding of newly learned task information also supports future learning and better attentional weighting in other tasks. Specifically, participants that continually learned three tasks in the Semantic-labeling group, versus the No-labeling group, showed a stronger relation in task performance between this continual task learning and subsequent learning of new reward contingencies in a value-based decision-making task. We also examined the impact of semantic labeling on reinforcement learning strategies and showed that participants in the Labeling group paid more equal attention to all stimulus features.

Concordant with earlier studies (Farashahi et al., 2017, 2018, 2020; Farashahi & Soltani, 2021), our results showed that people mostly adopt feature-based learning strategy at first, but gradually learn to also use an object-based learning strategy over time. Both groups appeared to show this shift to a similar degree. However, the Semantic-labeling group did show a more equal distribution of cognitive weights across features (antennae, leg, and mandible), compared to a higher weight assigned to the antennae feature in the No-labeling group. While speculative at this point, we believe this may hint at a potential upcoming shift toward an object-based strategy in the Semantic-labeling group. Namely, this balanced weighting of features suggests that participants in the labeling condition were paying equal attention to all features, rather than focusing on a single feature, and this approach may indicate that participants were more efficient at perceiving the stimuli as a unified object, rather than a collection of individual features. With prolonged training, this equal distribution of attention could have led to a more pronounced object-based learning strategy, where participants integrate all features into their decision-making process. Further supporting this, the results from the value-estimation task revealed that participants in the Semantic-labeling group provided significantly more accurate object-based value estimations compared to the No-labeling group. This finding suggests that labeling enhances participants’ ability to estimate the overall value of objects rather than relying on individual features.

An additional consideration is the potential influence of semantic labeling on reinforcement learning through a reduction in working memory load. Collins and Frank (2012) illustrated the link between working memory and reinforcement learning by demonstrating that performance is influenced by the number of stimuli participants must associate with a specific behavior. As the size of the stimulus set increased, participants’ performance declined, underscoring the impact of working memory limitations on reinforcement learning outcomes. In the same vein, recent research indicates that incorporating more nameable features can alleviate the decline in learning performance associated with larger sample sizes (Radulescu et al., 2022). Brady et al. (2016) demonstrated that individuals can store more information in greater detail when presented with real-world objects, independent of long-term memory. Similarly, Starr et al. (2020) showed that semantic knowledge directly enhances visual working memory, with familiar, nameable objects being easier to retain than unfamiliar, abstract objects, even for young children. In contrast, Yoo et al. (2023) suggested that a lack of semantic distinctness in stimuli can impair learning outcomes by directly affecting reinforcement learning processes rather than working memory. Overall, these findings suggest that existing knowledge and familiarity with objects can influence performance and reinforcement learning, either by expanding working memory capacity or by directly impacting reinforcement learning itself. Similarly, our findings showed that semantic labeling can improve reinforcement learning outcomes. Labeling may augment reinforcement learning by enhancing memory and familiarity with stimuli, allowing participants to assign value to each stimulus (object-based approach) easier rather than focusing solely on specific features, such as antennae in our case. Future studies could consider extending the reinforcement learning phase to further investigate how semantic labeling interacts with these cognitive systems to enhance learning.

In addition to the effects of working memory, attention also plays a critical role in reinforcement learning. By selectively processing relevant environmental cues, attention enhances behavioral efficiency and facilitates learning (Rmus et al., 2021). In parallel, research suggests that attention can be shaped by language. Lupyan et al. (2020) proposed that language and the nameability of stimuli influence attentional processes. Radulescu et al. (2022) further showed that nameability impacts how states are represented in reinforcement learning, with easier-to-name features helping individuals to more accurately identify task-relevant aspects that predict rewards. Considering these findings, the semantic labeling in our study may have guided participants’ attention toward features that were perceived as critical for determining future rewards. By providing labels, we likely influenced participants to allocate their cognitive resources evenly across all relevant features of the stimuli—such as the antennae, legs, and mandibles—optimizing their attention and enhancing both learning and decision-making processes.

Although our results show that language, specifically the use of semantic labels, plays a beneficial role in continual learning by reducing task interference, it is crucial to recognize that this benefit may not apply universally. Research suggests that verbal thinking can impede learning in certain tasks (Dijksterhuis, 2004; Liu et al., 2024; Schooler & Engstler-Schooler, 1990; van den Bos & Poletiek, 2008). For example, language may be less effective—or even disruptive—in tasks that require implicit statistical learning, those where verbal representation does not capture stimuli accurately, or tasks with labels that include exceptions to general rules. Therefore, caution is needed when extrapolating our findings to other learning domains, as some areas may benefit more from strategies that minimize language use.

There are, of course, also limitations to our results. First, while participants learned the three tasks with comparable success, the mean accuracy for the task associated with the mouth feature was lower than for the other two features, antenna and leg. This indicates that the saliency of the features was not equal, making it more challenging for participants to extract the mouth feature. Due to the lower salience of the mouth feature, we intentionally fixed the antenna feature as the informative dimension across participants in Experiment 3 to avoid confounding value-based decision-making results with differences in perceptual difficulty. Second, we utilized the same set of stimuli and semantic labels across all experiments. Using different stimuli or labels would enhance the generalizability of our results. Third, the imbalanced exclusion rates observed between Experiments 1 and 3 may be due to the unique nature of Experiment 3. This experiment involved two different paradigms, and data were collected from a different participant population with a one-year gap from Experiment 1 on Prolific. These factors may have contributed to the increased exclusion rate. Finally, we did not assess participants’ existing semantic knowledge prior to the experiments. Future studies could benefit from incorporating such assessments to better understand how pre-existing semantic knowledge may influence learning outcomes.

Future research could investigate the influence of semantic labels on task learning and the development of conceptual representations across different languages and cultures. A recent study found that languages that are geographically closer, historically related, or spoken by culturally similar groups tend to exhibit greater semantic alignment (Thompson et al., 2020). Furthermore, differences in labeling across languages may affect how concepts are acquired and represented semantically, leading to variations in perceptual interpretation (Lupyan et al., 2020). Understanding how these cultural and linguistic factors influence task representations and learning processes could provide valuable insights for both cognitive science and cross-cultural research. Additionally, exploring how enhancing or suppressing the semantic aspects of tasks influences task representations would be valuable. Future studies could examine the effects of presenting task cues (context) in humans where semantic knowledge is less well developed, such as children, to better understand how semantic information can facilitate learning and memory.

In this study, we examined the roles of semantic labeling and the sequence of stimulus and task cue presentations in shaping task representations and their impact on continual learning. Our findings suggest that semantic labeling supports the formation of more distinct task representations, thereby strengthening the organization and retrieval of task knowledge—an essential aspect of lifelong learning. These insights contribute to our understanding of the role of semantic anchors in human continual learning.

## ACKNOWLEDGMENTS

We would like to thank Tom Verguts for his helpful comments on an earlier version of this manuscript. This work was supported by an ERC Starting grant awarded to S.B. (European Union’s Horizon 2020 research and innovation program, Grant agreement 852570).

## FUNDING INFORMATION

This work was supported by an ERC Starting grant awarded to S.B. (European Union’s Horizon 2020 research and innovation program, grant agreement 852570).

## DATA AVAILABILITY STATEMENT

Data availability: https://osf.io/h6saz/.
